# Programmed cell death detection methods: a systematic review and a categorical comparison

**DOI:** 10.1007/s10495-022-01735-y

**Published:** 2022-06-17

**Authors:** Sana Kari, Kumar Subramanian, Ilenia Agata Altomonte, Akshaya Murugesan, Olli Yli-Harja, Meenakshisundaram Kandhavelu

**Affiliations:** 1 Molecular Signaling Lab, Faculty of Medicine and Health Technology, Tampere University, P.O. Box 553, 33101 Tampere, Finland; 2 Department of Biotechnology, Lady Doak College, Thallakulam, Madurai, 625002 India; 3 Institute for Systems Biology, 1441N 34th Street, Seattle, WA USA; 4 Computational Systems Biology Group, Faculty of Medicine and Health Technology, Tampere University, P.O. Box 553, 33101 Tampere, Finland

**Keywords:** Apoptosis, Necroptosis, Pyroptosis, Hallmarks, Biomarkers, Caspase, DNA fragmentation, ELISA, Immunohistochemistry, Electrophoresis

## Abstract

Programmed cell death is considered a key player in a variety of cellular processes that helps to regulate tissue growth, embryogenesis, cell turnover, immune response, and other biological processes. Among different types of cell death, apoptosis has been studied widely, especially in the field of cancer research to understand and analyse cellular mechanisms, and signaling pathways that control cell cycle arrest. Hallmarks of different types of cell death have been identified by following the patterns and events through microscopy. Identified biomarkers have also supported drug development to induce cell death in cancerous cells. There are various serological and microscopic techniques with advantages and limitations, that are available and are being utilized to detect and study the mechanism of cell death. The complexity of the mechanism and difficulties in distinguishing among different types of programmed cell death make it challenging to carry out the interventions and delay its progression. In this review, mechanisms of different forms of programmed cell death along with their conventional and unconventional methods of detection of have been critically reviewed systematically and categorized on the basis of morphological hallmarks and biomarkers to understand the principle, mechanism, application, advantages and disadvantages of each method. Furthermore, a very comprehensive comparative analysis has been drawn to highlight the most efficient and effective methods of detection of programmed cell death, helping researchers to make a reliable and prudent selection among the available methods of cell death assay. Conclusively, how programmed cell death detection methods can be improved and can provide information about distinctive stages of cell death detection have been discussed.

## Introduction

In 1972, John F.R. Kerr with his colleagues formulated the concept and coined the term apoptosis during his work on hepatocytes of rat [1]. Earlier in 1885, Walther Flemming precisely described the phenomenon of programmed cell death [2]. Apoptosis is a specific form of programmed cell death which occurs in multicellular organisms under normal physiological conditions to support different biological systems including normal cell turnover, immune system, tissue homeostasis, embryonic and brain development [3]. Apoptosis is the most organized and well-recognised program of eukaryotic cell death. Other than apoptosis, necroptosis, pyroptosis, and ferroptosis are different modes of cell death. However, apoptosis, necroptosis and pyroptosis are well-understood modalities of cell death which share some common and unique pathways and so far, they are best-studied forms of programmed cell death [4]. The process of apoptosis involving many regulatory sets of gene, not only begins after detection of any abnormality in cell function but also to control the rate of cell division [5]. When apoptosis is no longer required, the intracellular death program is activated, and cells commit suicide, as the number of cells in multicellular organisms is tightly regulated [6]. The occurrence of apoptosis displays morphological changes and triggering of biochemical cascade in the cells. These morphological changes include shrinkage and condensation of cells, increased density of cytoplasm and disassembling of nuclear envelop and formation of apoptotic bodies [7]. Such morphological changes are also considered as morphological hallmarks of apoptosis, while fragmentation of DNA, protein cleavage, and protein cross-linking as biochemical hallmarks of apoptosis [8]. The distinctive feature of apoptosis is non inflammatory and non-collateral damage cell death; wherein, cell death occurs without damaging neighbouring cells, making apoptosis a silent and immunologically inert process [5]. However, necroptosis and pyroptosis play the role of whistle blowers and initiate the release of alarmins and other proinflammatory signals into the cellular surroundings [4]. Necroptosis displays cytoplasmic swelling (oncosis), nuclear dehydration (pyknosis), dilation of golgi apparatus, mitochondria, endoplasmic reticulum, rupture of cell the membrane, vacuolation and spillage of cytoplasmic content on neighboring cells and considered as nonapoptotic form of cell death [9]. Secondary necrotic cells still exhibit some apoptotic features, such as condensed and fragmented nuclei (karyorhexis), loss of chromatin structure, and internucleosomal cleavage [10]. Difficulty in distinguishing between necroptotic and apoptotic cells makes the process of detection harder and challenging because both processes can occur simultaneously in the tissue or cell culture exposed to the stimulus [11]. In contrast, pyroptosis is characterized by rapid plasma membrane rupture, cytoplasmic swelling, and the release of proinflammatory intracellular contents. Pyroptosis is mechanistically and morphologically distinct from other forms of cell death stimulated by a range of microbial infections and non-infectious stimuli [12]. This review is an effort to categorise and summarise some conventional and non-conventional detection methods based on hallmarks and biomarkers of programmed cell death occurring due to intertwined cell death pathways of apoptosis, necroptosis and pyroptosis. All three modalities are so far, the most well understood and prevalent forms of cell death in multicellular organisms.

## Programmed cell death mechanism, crosstalk and its clinical importance

Cell-death inducing program usually begins via the same basic components either to terminate a cell due to developmental sequences or immune-mediate signals triggered by death receptors which is quite an extrinsic mode of cell death, and it is also considered as cell death by social control. On the contrary, when a cell encounters oxidative stress, viral infection, or injury inflicted by DNA damaging agent, mitochondrial cytochrome c-mediated intrinsic apoptotic pathway gets triggered which is also known as autonomous cell death [13]. In apoptosis, extrinsic pathway gets triggered when a ligand produced by natural killer cells or macrophages gets attached to death receptors, Tumor necrosis factor (TNF) receptor 1, or Toll-like receptors (TLRs) present on the cell surface, which recruit intracellular adaptor proteins (FADD, RIPK1 and procaspase-8) that bind and aggregate procaspase molecules to initiate proteolytic cascade. Mitochondria gets induced and starts releasing cytochrome-c into cytosol when the cell encounters any damage or stress. Cytochrome-c binds and activates adaptor protein (apoptotic protease activating factor 1, APAF1), which recruits procaspase-9 forming the apoptosome [14]. Both the extrinsic and intrinsic pathways work in a cooperative manner to ensure health and encounter the damage and removal of cells [5]. Overall, both apoptotic pathways are based on a set of highly specific cysteine-aspartic proteases known as caspases which are divided into two categories: initiator caspases (− 2, − 6, − 8, and − 10) and executioner caspases (− 3, − 6, and − 7) [15]. This protease family possesses a cysteine at the active site and cleaves the specific aspartic acid site of the target protein. Caspases are synthesised in the cell and remain inactive and are called procaspases, which eventually get activated by cleavage at the aspartic acid site by other caspase and initiate a series of proteolytic cascade. This proteolytic cascade is a non-reversible, destructive, and self-amplifying cascade which once started, does not turnback until it terminates the cell [6]. Regulation and activation of procaspases are partially supported by Bcl-2 family of intracellular proteins. B cell lymphoma 2 (Bcl-2) is a protooncogene which, produces pro- and anti-apoptotic proteins [16]. In 1988, David L. Vaux and is colleagues discovered the anti-apoptotic and tumorigenic role of Bcl-2 [17]. Bcl-2 family of proteins is considered a powerful regulator, which regulates apoptosis through mitochondria in an apoptotic cell [18]. From Bcl-2 family, Bcl-XL, which is a homologue of Bcl-2, interacts with APAF1 to inhibit the activation of associated caspases. Likewise, Bcl-2 itself inhibits apoptosis by partially blocking the release of cytochrome c from mitochondria and maintains membrane homeostasis [19, 20]. However, other members of Bcl-2 family like Bax and Bak, promote procaspase activation and cell death instead of inhibiting cell death [6]. It has been observed that heterodimerisation of anti-apoptotic and pro-apoptotic members of Bcl-2 family promotes cell death [21, 22]. Other than Bcl-2 family, IAP (inhibitor of apoptosis) is another very important family of intracellular proteins, which has a central role in regulating apoptosis. IAP inhibits apoptosis by binding with procaspases and hinders their activation, as well as with some particular caspases to hinder their entire activity [6]. Cell death pathways are tightly connected, and cross regulate each other. Components involved in apoptosis also participate in necroptosis. Caspase-8 has been identified as a crucial regulator of necroptotic and apoptotic pathways. Preventing the formation of necrosome by switching the cleavage of RIPK1 and RIPK3 (Receptor-interacting serine/threonine-protein kinase), favors apoptosis over necroptosis. Additional inhibition of caspase-8 by microbes or pharmacological agents triggers the necroptotic pathway. Activation of cellular receptors triggers necroptosis [23]. Almost similar death receptors are involved in the execution of necroptosis, which are involved in apoptosis, for example TLRs, Fas/FasL [24]. Upon activation of receptors, adaptor proteins are recruited which interact with caspase-8 and RIPK1, which further recruits RIPK3. RIPK1/RIPK3 complex recruits and phosphorylates MLKL (mixed lineage kinase domain-like) and forms necrosomes. MLKL oligomers form large pores in the plasma membrane and lead to necroptotic cell death [4]. A recent study by Yang et al. revealed that RIPK3 directly phosphorylates and activates the E3 subunit of the pyruvate dehydrogenase complex and promotes aerobic respiration and mitochondrial ROS production [25]. The involvement of caspase has also been identified in the mechanism of pyroptosis. Caspase-1 dependence is a unique feature of pyroptosis. However, caspase-1 is not involved in the apoptotic pathway of cell death and caspase-1 deficient mice showed no defects in apoptosis [12]. Furthermore, none of the initiator and executioner caspases of apoptotic pathways have been found involved in pyroptosis. Pyroptosis is an inflammatory form of cell death triggered by intracellular sensors like NLRP3 that detect DAMPs (damage-associated molecular pattern), PAMPs (pathogen-associated molecular pattern), osmotic imbalance, membrane disturbance, and ion efflux [4]. PAMPs are derived from microorganisms and trigger inflammation in response to an infection [26]. Lipopolysaccharide (LPS) is one of the well-known PAMP found in outer cell wall of gram-negative bacteria [27]. DAMPs are derived from host cells including dead or dying cells, tumour cells, or products released from cells during stressful conditions [26]. DAMPs are also known as sterile inflammatory responses as they are derived from the host material. DAMPs and PAMPs bind to pattern recognition receptors (PRR) like TLRs, NLRs and are absent in melanoma 2-like receptors (AIM2) [27, 28]. PRR is expressed by immune cells like macrophages, monocytes, dendritic cells, and mast cells [26, 29].

**Fig. 1:**
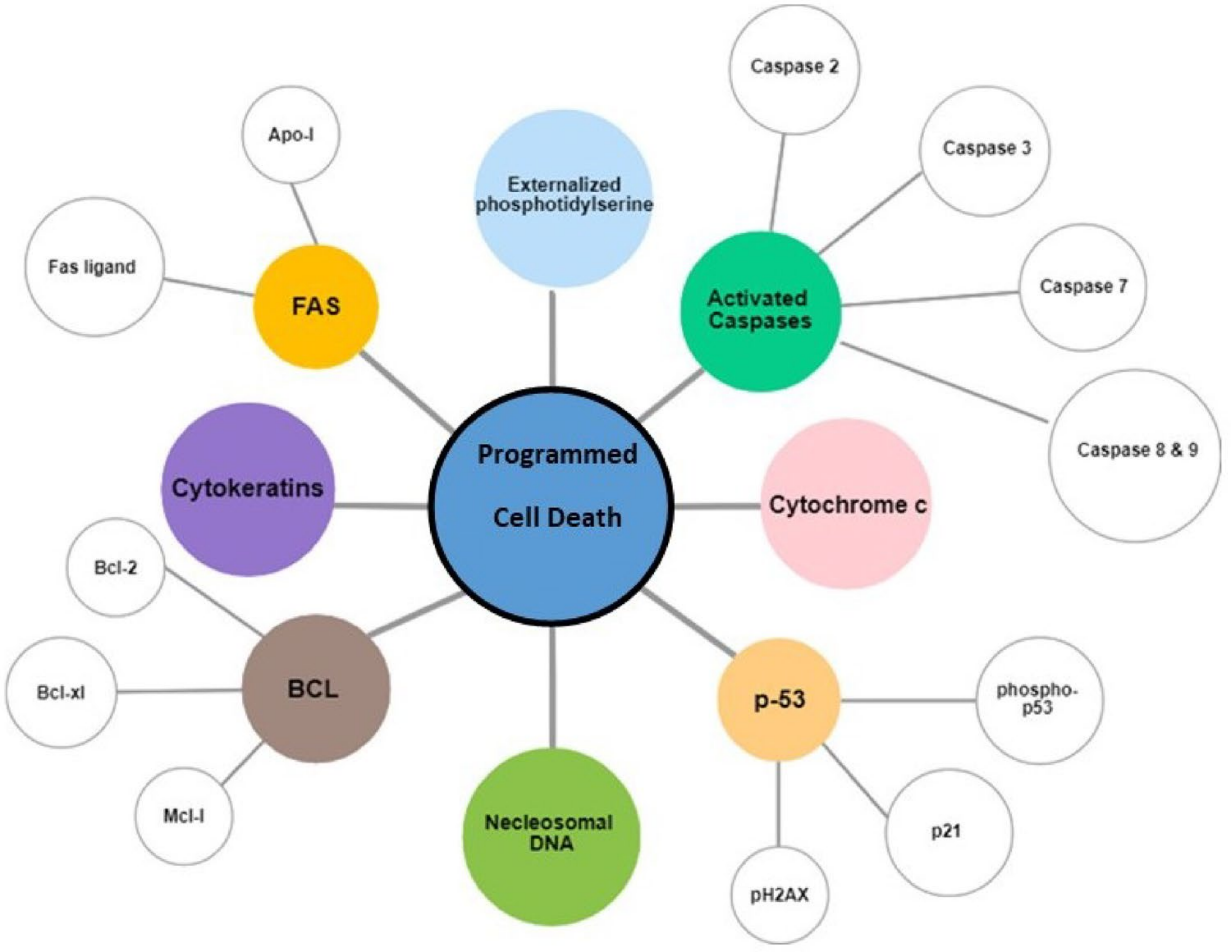
Biomarkers of programmed cell death that can be measured in the blood and tissues through different techniques which indicate the occurrence or onset of programmed cell death

In pyroptosis, active sensors recruit the adapter ASC, which forms a micron-sized structure called the inflammasome. These inflammasomes act as a platform for the activation of caspase-1, which consequently activates gasdermin D, which is responsible for pore formation in the plasma membrane and initiates the release of mature IL-1β and IL-18 causing “ballooning effect” and pyroptosis. RIPK3 has been found critical in the activation of NLRP3, which indicates cross-regulation of necroptosis and pyroptosis. RIPK3 regulates the caspase-8 activity and the activation of NLRP3 of TLR or TNFR1/2. Activation of NLRP3 and secretion of IL-β is also connected to downstream of the intrinsic pathway of apoptosis i.e., BAX/BAK-dependent mitochondrial destabilisation followed by caspase-3/7 activation [4]. Understanding cell death mechanism is very vital to combat diseases through drug development and it is very important to provide proof of mechanism for drug development, specifically in the clinical area. Different components of cell death cascade are potential biomarkers of programmed cell death as shown in Fig. 1. Biomarkers of programmed cell death can be exploited as the best opportunity for drug discovery and clinical trials, with the help of available genomic tools and technology [30]. Several drugs have been developed to inhibit anti-apoptotic proteins and enzymes of the Bcl-2 family [5]. Malfunction in death machinery can be a direct or indirect reason of various diseases. Dysregulation of programmed cell death usually appears in the form of proliferative and degenerative diseases. Insufficient or excessive occurrence of cell death, inaccurate timing, and location of apoptotic death are also different aspects of diseases [31]. It has been proved that several types of cancer exist because of defects in the cell death machinery. Increased apoptosis is a typical trait of AIDS [3]. p53 mutation and inhibition of apoptosis also cause cancer. For example, cervical cancer is triggered by the viral inhibition of apoptosis. Likewise, an excessive rate of apoptosis cause tissue atrophy. Alzheimer’s disease, Parkinson’s disease and amyotrophic lateral sclerosis (ALS), ischemic injury after myocardial infarction and stroke are the classic examples of elevated rates of apoptosis [7]. Similarly, RIPK1 and RIPK3, which are components of necroptosis have been found involved in the development of inflammatory disease such as rheumatoid arthritis, multiple sclerosis, and Crohn’s disease because necroptosis plays a considerable role in the induction of inflammation [32].

## Categorisation of programmed cell death detection methods

It is very important to analyse cell death processes which determine the cell death rate and sensitivity. Conventionally, cell survival, clonogenic, and membrane permeability assays are used to examine the cell death rate. Colorimetric substrate is used to assess metabolic activity such as mitochondrial succinate dehydrogenase activity which depicts cell survival and is considered cell survival assay. However, clonogenic assays typically stain colonies of cells that proliferate from cells that are resistant to or recovered from a cell death challenge and retain proliferation potential. That is why, cells arrested in the G_1_ or G_2_ phase cannot be examined through clonogenic assays although the cells do not undergo cell death. Cell death, which is induced via drugs or targeted genes, may affect metabolism or proliferation without inducing death, and such a condition sometimes leads to misinterpretation using metabolic readouts without actually reflecting cell death induction. Alternatively, membrane permeability assays are used which are considered more reliable as they measure the end stage of the cell death process itself. Following are a few programmed cell death detection methods, which are categorised based on morphological and biochemical hallmarks. The principle, measurement method, specificity and application of each method have been described briefly for better understanding.

▪ Membrane permeability/damage detection methods
  1. Annexin V binding assay
  2. Lactate dehydrogenase assay
  3. Electrochemical methods
▪ Mitochondrial damage/alteration detection methods
  1. MTT and XTT assay
  2. Mitochondrial membrane potential detection
  3. Mitochondrial activity of streptolysin O permeabilised cells assay
  4. Cytochrome c release detection
▪ Caspase activity detection methods
  1. ELISA
  2. Fluorometric and colorimetric assays
  3. Immunohistochemical methods
  4. Laser and mass spectroscopic methods
▪ p53 activity detection methods
  1. FASAY
  2. p53 protein analysis methods
▪ DNA fragmentation/denaturation/condensation detection methods
  1. APO ssDNA assay
  2. TUNEL assay
  3. ISEL
  4. ELISA
  5. Gel electrophoresis-based methods
  6. DNA-specific fluorochrome based methods

## Membrane permeability/damage detection methods

An increase in cell membrane permeability is the onset of programmed cell death. The cell starts shrinking/swelling, which alters the permeability and morphology of the cell membrane. To detect an alteration in permeability and morphology of the cell membrane, the following detection methods are used.

### Annexin V binding assay

**Fig. 2:**
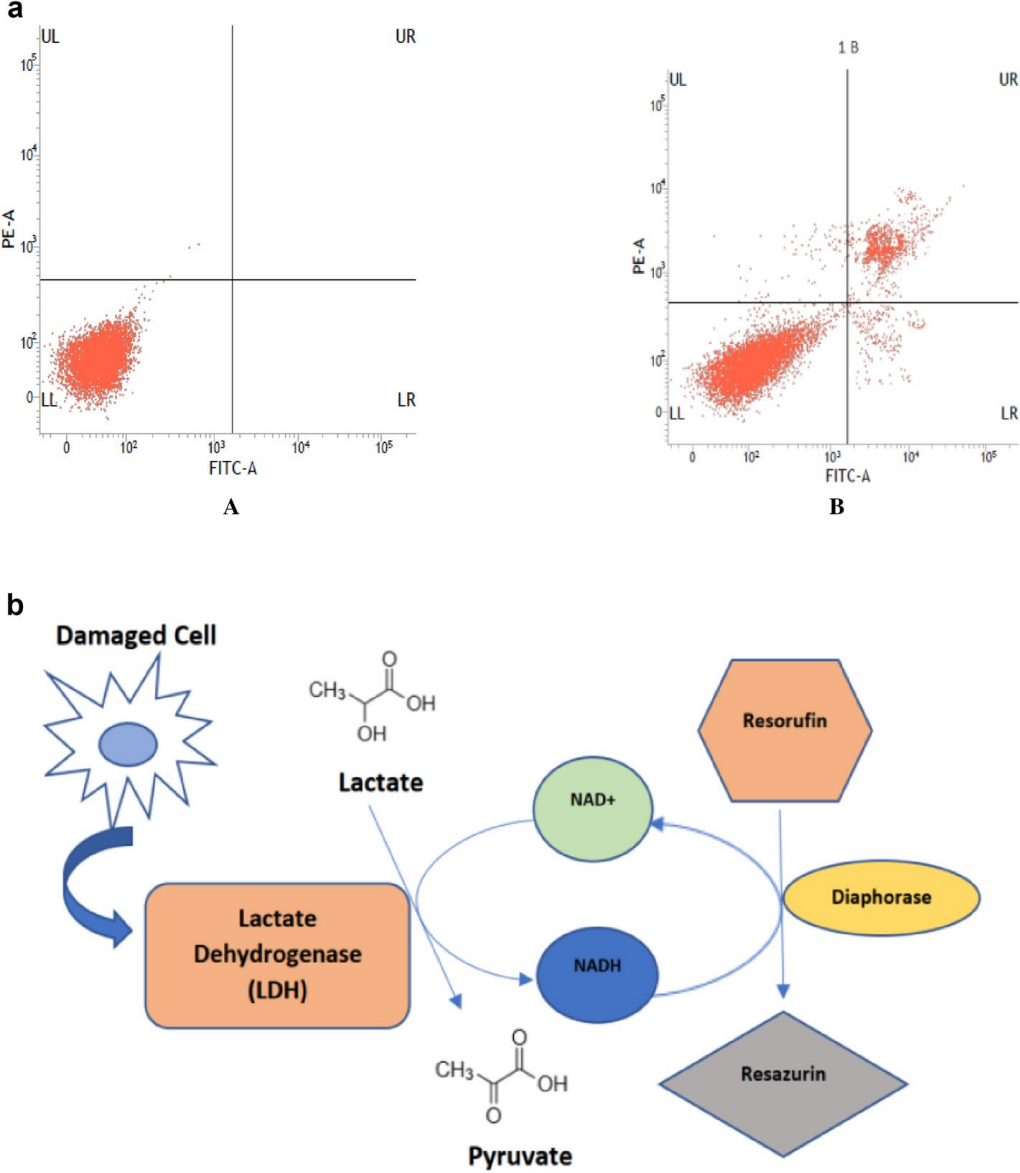
**a** FACS assessment of cell apoptosis using Annexin/PI staining (A) untreated MCF-7 cells (B) HNPMI (an indoline derivative) treated MCF-7 cells, UL—Necrotic cells, UR—Late apoptotic cells, LL—Viable cells, LR—Early apoptotic cells [39]. **b** Fluorometric quantification of released cellular lactate dehydrogenase (LDH) when cells are damaged or under stress. LDH released into the culture medium is measured with a diaphorase coupled enzymatic assay that results in the conversion of a non-fluorescent compound (resazurin) to a fluorescent compound (resorufin) measured by a fluorometer. The assay detects even low-level damage to the cell membrane, which is not detectable with other methods [45]

Annexin V binding assay is mainly an established technique for the identification and quantification of apoptotic cells. Annexin V is a calcium-dependent phospholipid-binding protein which has a high affinity for phosphatidylserine (PS). A healthy cell surface is protected with lipids distributed asymmetrically on the inner and outer leaflets of the membrane of the plasma [33]. PS is located on the cytosolic side of the plasma membrane in healthy cells. The onset of apoptosis which can occur through the cytotoxic compound dislocates the PS and translocates it to the extracellular leaflet of the membrane. This dislocation of PS is detected through fluorescently labelled Annexin V, which conjugates with externalised PS [9]. Annexin V is usually used in conjugation with 7-amino-actinomycin (7-ADD) or propidium iodide (PI) which can only bind to nucleic acids and create a clear difference of apoptotic stage [34]. Annexin V/PI protocol is a commonly used approach for studying apoptotic cells. PI stains only dead and late apoptotic cells because it only enters the cells with compromised permeability of the membrane [35, 36]. PI intercalates into nucleic acid and displays red fluorescence [36]. Apoptotic cells appear single positive for Annexin V staining via flow cytometry. However, due to increased membrane permeability in the pyroptotic cells, PI and Annexin V are free to enter the cell to stain PS and DNA, which exhibits a double-positive staining for PI and Annexin V. Double-positive stained cells can also be marked as necrotic cells due to membrane rupture [37]. The phenomena of binding calcium-dependent Annexin V, which shows a high affinity for PS residues can be performed by using commercially available kits and can be observed through flow cytometry (Fig. 2a), fluorescence, or light microscopy [33, 38].

### Lactate dehydrogenase assay

Lactate dehydrogenase (LDH) appears in the outer space of cell when the cell membrane permeability is compromised, or the cell membrane is damaged. LDH is a cytosolic enzyme present in all living cells and only appears in the extracellular environment because of cell damage due to necroptosis and pyroptosis. In pyroptosis, activated caspase-1 cleaves gasdermin d, which releases the N-terminal pore-forming domain, which gets inserted into the plasma membrane and triggers osmotic lysis. Lysis releases cytoplasmic contents including the LDH enzyme [40]. Tumour necrosis factor (TNF) family members trigger the process of necrosis with the help of Fas and TRAIL receptor, which is considered a potential form of programmed cell death [41]. The LDH assay can also be used to measure TNF activity in different cells [42]. There are five different isoenzymes of LDH but all of them catalyse the same reaction, which is a reduction of NAD + to NADH, H + , and oxidation of lactate to pyruvate [9]. The LDH assay can be used to determine cell viability and cell proliferation. It is a simple, quick, and accurate method to determine cell proliferation [43]. The LDH assay is both a colorimetric assay and fluorometric assay. In colorimetric assay, a tetrazolium salt TTC (2,3,5-triphenyl-2H-tetrazolium chloride) detects the cytotoxicity of a compound/drug. The conversion of white TTC into red TPF (1,3,5-triphenylformazan) occurs with the help of NADH and H + . The amount of color produced is measured by standard spectroscopy at 492 nm and its proportional to the number of damaged cells. The fluorometric assay of LDH is based on the conversion of non-fluorescent resazurin to a fluorescent compound resorufin and the same reaction takes place, which is the conversion of lactate into pyruvate (Fig. 2b). The amount of light absorbed is directly proportional to cells with compromised membrane permeability or lysed cells. Therefore, early LDH measurement is indicative of pyroptotic cell death. The levels of LHD in a sample of cell lysate are measured with commercially available LDH assay kits. The LDH assay detects low-level damage to the cell membrane and does not damage the population of healthy cells and can be performed directly in the cell culture wells [44].

### Electrochemical methods

Detection of programmed cell death does not always require complex and expensive instrumentation. Electrochemical devices are evolving in the field of research. There are different kinds of electrochemical devices available for the detection of programmed cell death. Liu et al., (2009) designed a pyrolytic graphite electrode modified with polyethyleneimine and Annexin V. The purpose of adding Annexin V in polyethyleneimine (PEI) film is to retain its affinity for PS. The binding of calcium-dependent Annexin V to the cells in the electrode inhibits redox reaction of [Ru(Nh3)5Cl]2 + / + at the electrode surface. [Ru(Nh3)5Cl]2 + / + is used as an electrochemical probe, which depicts a good electrochemical response. The electrochemical probe shows changes in cyclic voltammograms after interaction between Annexin V and externalised PS that indicates the presence of apoptotic cells in solution [46].

The dielectrophoretic (DEP) assay is another electrochemical approach that is conducted by a 3DEP cell analyser or other DEP assay system. The DEP assay is used to measure membrane capacitance and cytoplasmic conductivity during apoptosis. DEP is a non-invasive assay of apoptosis detection which does not require labels or markers. Dielectrophoresis detects apoptosis more rapidly than other methods and also conclusively discriminates between apoptotic and non-apoptotic cells. DEP is an effective tool for quantifying cell death and provides results which are comparable to standard assay such as MTT, flowcytometry and trypan blue [47]. Lv et al., (2013) induced apoptosis in NB4 cells by using cytosine arabinoside (Ara-C) and investigated apoptosis through DEP assay. The DEP system used in the assay consisted of a chip made up of non-closed ring gold electrodes placed on the surface of a clean glass wafer using standard photolithography. Sine signals were applied to the chip signal generator in the range of 10–15 MHz. Measurements were observed through a CCD camera coupled to a fluorescence microscope. DEP measurements were made by determining crossover frequencies, which were interpreted in determining membrane capacitance and cytoplasmic conductivity. The group observed decreased membrane capacitance and cytoplasmic conductivity, which indicated altered dielectric property of the cells. Membrane capacitance depends on plasma membrane surface morphology and an alteration in morphology indicates the increase or decrease in membrane capacitance. The DEP analysis also indicated a decrease of intracellular potassium and increased intracellular calcium and sodium level that made apoptotic cells amenable for characterisation by using DEP analysis [47].

## Mitochondrial damage/alteration detection methods

Alteration in mitochondrial activity and release of cytochrome c are considered signs of apoptosis and other programmed cell death. Mitochondrial dysfunction induces cell death and it has been identified as a center of programmed cell death pathways. Mitochondria undergo various changes which indicates cytotoxicity of the cell. The following detection methods indicate alteration/damage in the mitochondria during programmed cell death.

### MTT and XTT assay

MTT (3-4,5-dimethylthiazol-2-yl)-2,5-diphenyltetrazolium bromide) and XTT (2,3-bis(2-mehtoxy-4-nitro-5-sulfophenyl)-2H-tetrazolium-5-carboxanilide) are tetrazolium salts. Unlike TTC, MTT and XTT are used to detect viable cells among dead cells and they both are used to measure the cytotoxicity of a drug/compound [48–51]. TTC enters only in damaged cells while MTT and XTT produce insoluble and soluble formazan, respectively. MTT serves to assess the energy capacity of a cell indirectly and it is dependent on the mitochondrial respiration of a cell [52]. MTT is a yellow tetrazolium salt, which gets reduced to purple formazan crystals with the help of the succinate dehydrogenase enzyme present in mitochondria which indicates the activity of mitochondria [53]. The formation of purple formazan exhibits as a marker of active metabolism of viable cells. The darker the solution, the greater the number of metabolically active viable cells [54]. As mentioned earlier, MTT produces insoluble formazan, gets accumulated in the cells and near the cell surface, which is solubilised before measuring absorbance through a multi-well spectrophotometer at 570 nm. MTT assay is a non-radioactive time-dependent assay. Longer incubation time results in the accumulation of crystalised formazan and increased sensitivity. However, incubation is limited due to the cytotoxicity of reagents which are used to detect signals [55]. MTT assay has been widely used and is considered a gold standard for measuring cell viability and drug toxicity. However, MTT has been proven nonspecific and inconsistent in many experimental circumstances [56]. The XTT assay is based on the second generation of colourless tetrazolium salt, an improved version of MTT, in which solubilised formazan products are produced and measured directly after incubation. Nevertheless, there is an increase in negative charge on tetrazolium reagents that also decreases their ability to pass across cell membranes. In conjunction with intermediate electron acceptance reagents such as phenazine methyl sulphate (PMS) or phenazine ethyl sulphate (PES), these molecules enter viable cells, shrink into the cytoplasm or cell surface, and exit cells where tetrazolium can be converted into the soluble product of formazanine [57].

### Mitochondrial membrane potential detection

**Fig. 3:**
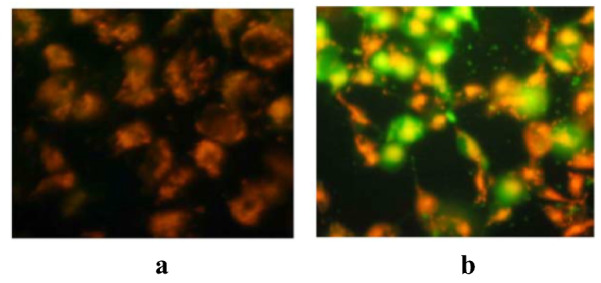
**a** JC-1 dye accumulates in the mitochondria of healthy cells as aggregates (red–orange fluorescing). **b** Due to collapse of mitochondrial membrane potential in the treated cells, the JC-1 dye remains in the cytoplasm in the monomeric form, which fluoresces green [61]

The loss of mitochondrial membrane potential (MMP), which is symbolically represented as Δψm, may not be an early sign of cell death, but it is a consequence of the death signaling pathway. The opening of mitochondrial permeability transition pore induces depolarisation of transmembrane potential, the release of apoptotic factors like AIF (apoptosis-inducing factor) and loss of oxidative phosphorylation. The release of cytochrome c may not be dependent on MMP, but the release of AIF is. Mitochondrial permeability transition pore (PTP) is a mega-channel, which is thought to be the source of release of different apoptogenic factors and cause of mitochondrial transmembrane potential (TMP) [58]. To detect the MMP and TMP, there are several membrane-permeable lipophilic and cationic fluorescent dyes available such as 3,3’-dihexylyoxacarbocyanine iodide [DiOC6(3)], rhodamine-123 (Rh123), tetramethyl rhodamine methyl and ethyl easter (TMRM and TMRE), 5′6′,6′tetracholor-1,1′,3,3-tetraethylbenzimidazolecarbocyannine (JC-1) and cholormethyl-X-rosamine (CMX-Ros) [59, 60]. All these dyes can be used qualitatively in fluorescence microscopy or quantitively in flow cytometry or microplate spectrophotometry. When apoptosis begins and the cell becomes unhealthy, and consequently, mitochondrial outer membrane loses its electrochemical gradient and cationic dye starts diffusing into the cytoplasm and emits fluorescence (usually green), which is different from the aggregated form that accumulates in the mitochondria when it is active (usually red) as shown in Figure 3. The red/green fluorescence ratio is used to determine the cell mitochondrial function [15, 59]. The inner side of the inner mitochondrial membrane is negatively charged that stores the cationic fluorochromes in the mitochondria [36]. These dyes are used with different conjugates such as PI and FCCP to make a clear difference between necrotic and apoptotic cell death and to discriminate the different stages of apoptosis. Table 1 shows the list of fluorochromes/fluorescent dyes which detect TMP and MMP through flow cytometry at 400–600 nm.

**Table 1:** Fluorescent dyes used in flow cytometry to detect changes in MMP

S. No.	Fluorescent dyes	Fluorescence	Indication
1	DiOC_6_(3)	Green	Indicates acute changes in plasma membrane and MMP
2	Rh123	Green	Indicates changes in plasma membrane and MMP
3	TMRM	Red–Orange	Indicates high or low MMP
4	JC-1	Red/Green	Indicates high or low MMP
5	CMX-Ros	Red	Indicates loss of MMP

### Mitochondrial activity of streptolysin O permeabilised cells assay

The key physiological function of mitochondria is the production of energy by oxidative phosphorylation of adenosine triphosphate (ATP), oxygen is used to promote mitochondrial respiration, which in turn is used to create a proton gradient across the inner mitochondrial membrane. The proton gradient is also called as the potential gradient of the mitochondrial transmembrane, which then drives many mitochondrial functions, such as ATP synthesis, transport of calcium and other ion exchangers, and import of proteins [62]. Additional mitochondrial functions are the production and detoxification of reactive oxygen species (ROS), involvement in the forms of apoptosis, synthesis and catabolism of metabolites, control of cytoplasmic and mitochondrial matrix. Mitochondrial dysfunction may be caused by anomalies in either of the above-mentioned systems [63]. In mitochondrial bioenergetics, the proton circuit is central, so improvements in the ability of the mitochondrial membrane have an immediate effect on ATP synthesis. Treatment of cells with streptolysin O on ice is a simple way of measuring mitochondrial ATP synthesis to make plasma membranes permeable without harming mitochondrial function. The assay is called mitochondrial activity of streptolysin O permeabilised cells (MASC) assay and it is a simple, rapid, and sensitive measurement of ATP synthesis. MASC assay measures the loss of mitochondrial ATP synthesis which occurs due to cellular dysfunction. Cells in a microplate are exposed to streptolysin and then subsequently washed to remove the excess of streptolysin O, shifting the temperature to 37 °C. After the addition of an inhibitor of adenylate kinase, Ap5A, the reaction is initiated by supplementing ADP, a substrate for respiratory chain and luciferin/luciferase. It is easy to track ATP synthesis with a microplate luminometer [64]. The ATP Assay Kit-Luminescence enables the quantitation of intracellular ATP by luciferase luminescence assay. MASC assay is non-specific for detecting apoptosis as it only indicates the loss of mitochondrial ATP synthesis, which is not the only reason for the onset of apoptosis or any other programmed cell death.

### Cytochrome c release detection

During apoptosis, cytochrome c translocates from the mitochondria to the cytosol, and interacts with APAF1 and recruits pro-caspase 9 into a multiprotein complex with cytochrome c and APAF1 called the apoptosome. The translocation of cytochrome c from the mitochondria to the cytosol is a critical process and it activates the cascade of caspase, which leads to programmed cell death or apoptosis. On the contrary, cytochrome c could be released in the extracellular space due to cell damage and it may serve as a DAMP. Inappropriate translocation of cytochrome c decides anti-inflammatory or pro-inflammatory properties of cytochrome c. Cytochromes c is among such molecules which trigger inflammation once translocated in the extracellular space [65]. Cytochrome c leaves the cell and reaches the serum of patients suffering from cancer and undergoing cancer therapy. Cytochrome c is also released into blood circulation, following myocardial infarction, resuscitation, and cardiac arrest. During such events, cells die and released cytochrome c gets mixed with blood circulation, and serves as a marker for mitochondrial injury and organ damage. It has been observed that the level of cytochrome c in serum is higher in patients with systematic inflammatory response and multiorgan dysfunction syndrome [66].

The release of cytochrome c is currently the best-studied event because its release from the mitochondria is an important step for apoptosome formation and the progression of cell death processes [67]. The release of cytochrome c occurs due to oxidative stress, UV radiation, or chemotherapeutic drug [66]. Detection of the release of cytochrome c is very important as it not only provides valuable information about the nature and extent of apoptosis, but also serves as a preclinical indicator of various pathologies, therapeutic treatment, and medical diagnostics [66]. Measurement can be done by using different techniques such as, flow cytometry, ELISA, HPLC, spectrophotometry, and western blotting.

For flow cytometry, cultured cells are fixed and incubated with anti-cytochrome c monoclonal antibody overnight and are washed with blocking buffer. Alexa 488 is used as a fluorochrome which detects the release of cytochrome c from mitochondria by conjugating with anti-cytochrome c monoclonal antibody and displays a relatively smaller peak/fluorescence as compare to the fluorescence of intact mitochondria. This technique offers valid assays for cytochrome *c* release during apoptosis. The assay has the ability to rapidly and non-subjectively quantitate the number of cells that have cytoplasmic cytochrome c. This rapid assay can be used on a variety of adherent and nonadherent cell lines and it is an apoptosis-specific assay [68].

**Fig. 4:**
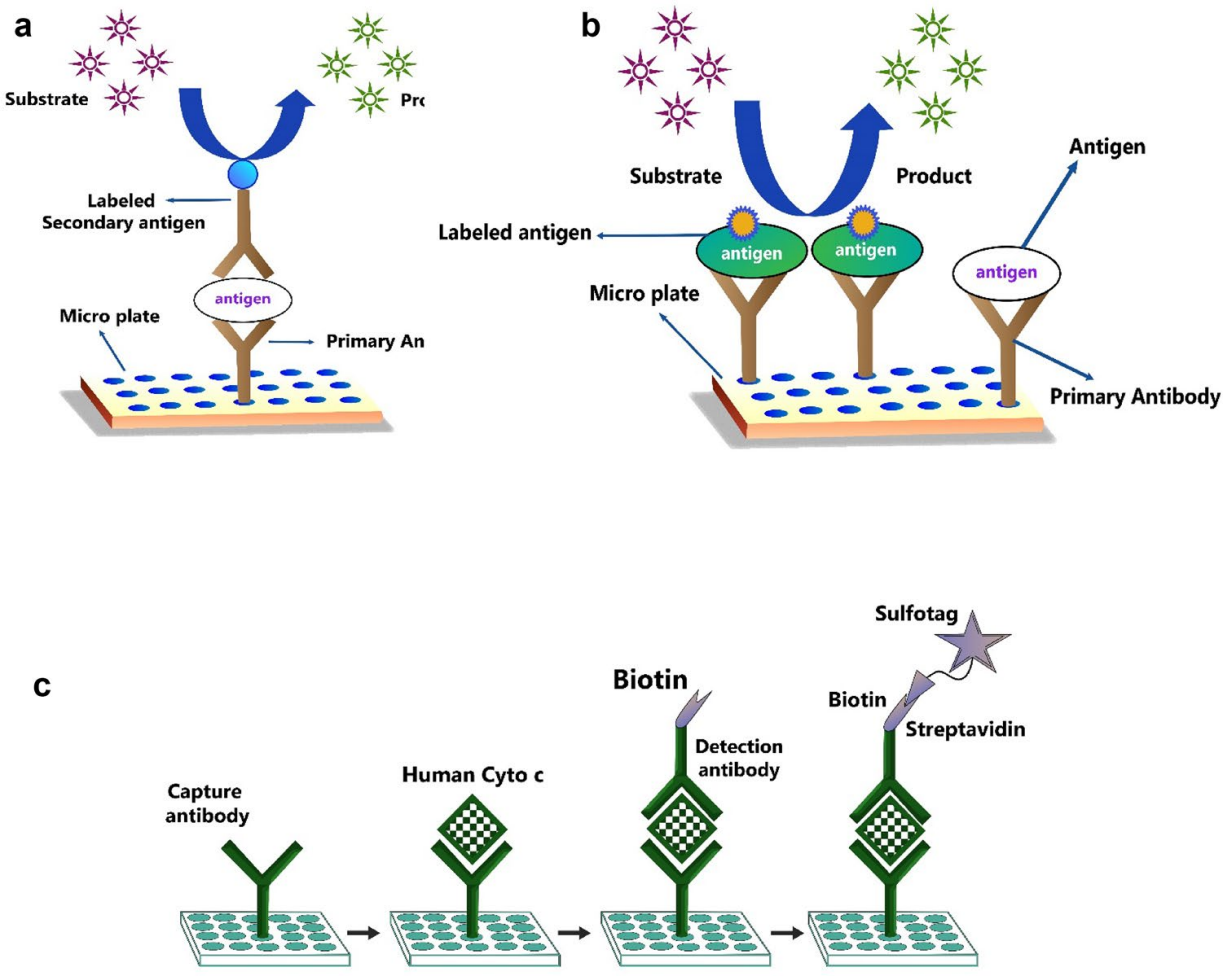
**a** Schematic representation of sandwich ELISA. **b** Schematic representation of competitive ELISA. **c** Schematic representation of human ECL indicating electrochemiluminescence

Other than flow cytometry, ELISA and western blotting are most commonly used for detecting the release of cytochrome c. There are few types of ELISA assays available for detecting the release of cytochrome c in the serum as it is an immunological assay, and is used extensively and specifically for the diagnosis of disease and biochemical research. Among those techniques, the Sandwich immunoassay of ELISA technique is broadly used. The assay is executed by coating the surface of the microplate with a constant amount of primary antibody specific to cytochrome c, as shown in Fig. 4a. In the next step, a series of dilutions of cytochrome c standard is added to antibodies on the microplate surface. Unbound antibodies are removed by washing and subsequently, enzyme-linked secondary antibody specific to cytochrome c is added and again the washing step is performed to remove unbound enzyme-labelled secondary antibodies. A substrate solution is added to the wells and the colour is developed, which is in proportion to the amount of cytochrome c bound to the antibody. Development of colour is stopped by adding stop solution and the intensity of the colour is measured by photometer at 400–600 nm. The high intensity of colour depicts high concentration of cytochrome c in the sample and indicates pathology. This technique is not apoptosis-specific but is generally used for estimating cell death burden in response to therapy [66].

On the contrary, competitive immunoassay of ELISA technique is based on only single type of antibody specific to the labelled cytochrome c, which competes with unlabelled cytochrome c to bind with the coated antibody, as shown in Fig. 4b. The colorimetric quantification of antibody-bound labelled antigen is done by interpreting the intensity of colour. The intensity of the colour is inversely proportional to the amount of free antigen in the sample [66].

Another type of ELISA which is used to detect the release of cytochrome c is electrochemiluminescence-ELISA (ECL-ELISA). It is a quantitative method for the measurement of antigen or antibody, based on the ECL signal before and after immunoreaction. In this method, microplates with carbon electrodes are integrated to the bottom of each well. The assay involves the same method of antibody coating and targeting protein. The unique step is the addition of ECL-labelled streptavidin sulfo-tag, which makes it different from other ELISA techniques. The sulfo-tag is an N-hydroxy-succinimide ester, which conjugates directly to the biotinylated detection antibody (Fig. 4c). A buffer containing electron carrier ‘tripropylamine’ is added and the plate is loaded into Meso Scale Discovery (MSD) imager. The imager supplies voltage to the electrodes of the microplate and sulfo-tag emits the light. The imager measures the intensity of the emitted light, which represents the quantitative data of the target protein level [69]. ECL-ELISA is not an apoptosis-specific assay, and is used for identification of biomarkers associated with various disease stages and/or protection against diseases [70].

Western blot is regarded as a classic technique for protein detection. It allows specific identification and characterisation of proteins, and specifically detects proteins involved in programmed cell death. It is able to distinguish the type of cell death i.e., apoptosis, necroptosis, etc. Western blot also detects the modification of proteins related to programmed cell death. For instance, the phosphorylation of RIPK1, which modulates apoptosis and necroptosis can be analysed through western blotting with the help of special antibodies [71]. Detection of cytochrome c through western blot involves electrophoretic sifting which is followed by immunoassay. Like ELISA, cytochrome c specific antibody is used in western blot as well. There are many kits available commercially to perform western blot for the detection of cytochrome c.

## Caspase activity detection methods

Apoptosis might begin via intrinsic or extrinsic pathway but converges on the execution pathway, which is initiated by cleavage of caspase-3. Likewise, pyroptosis is also a caspase-dependent form of cell death. Once caspases are activated, the proteolytic cascade begins, and it is considered an irreversible process. This proteolytic cascade amplifies cell death signaling pathways and leads to speedy cell death [10]. Following are the methods that are used to detect caspase activity in response to cell death.

### ELISA

**Fig. 5:**
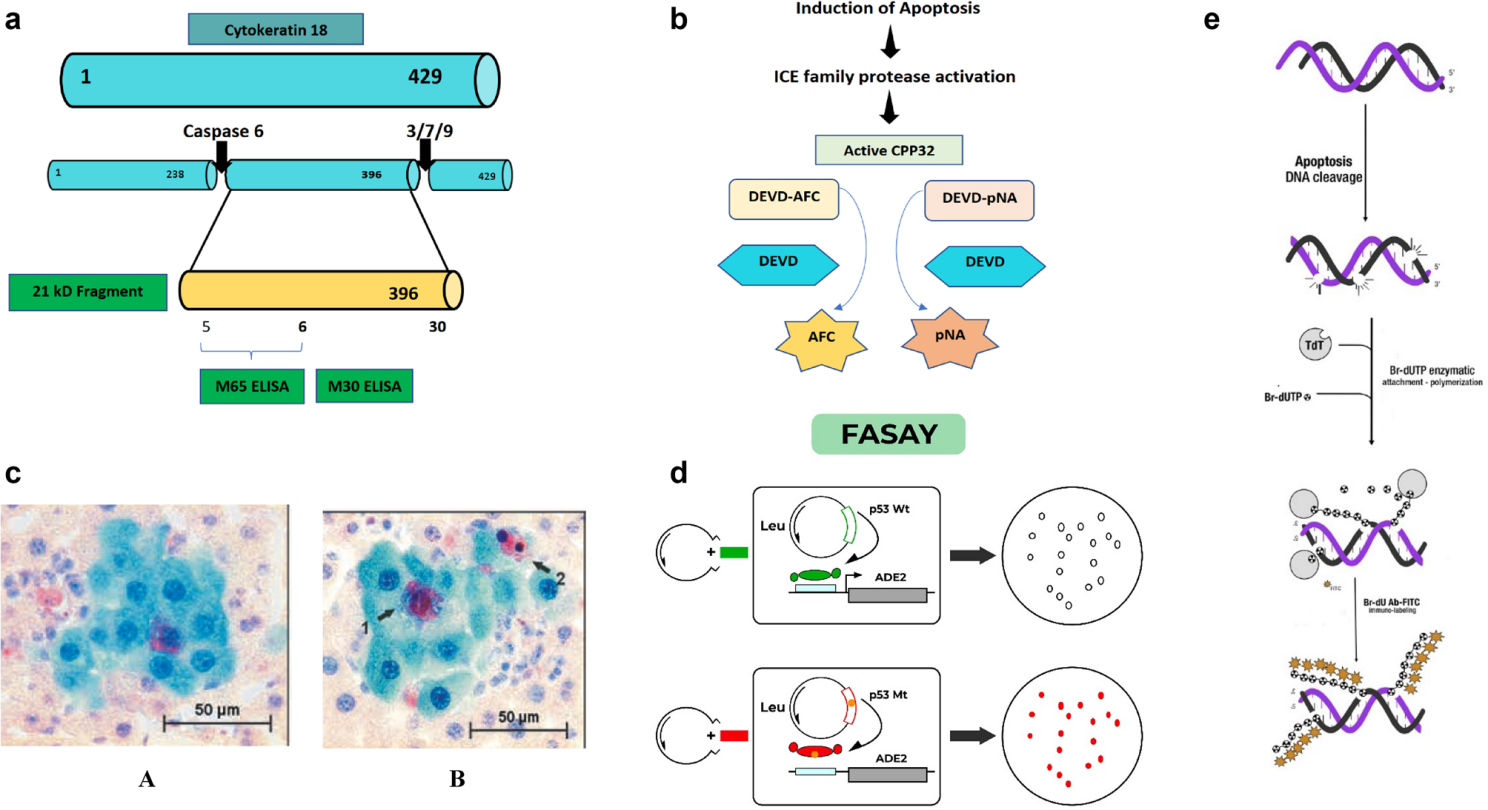
**a** Schematic diagram of CK18 epitope map targeted by antibodies which are utilised in M30 and M65 sandwich ELISA assays. In M65 ELISA, M5 and M6 are used as detector and catcher antibodies, respectively. However, in M30 ELISA assay, M5 is the catcher and horse radish peroxidase-conjugated M30 is the detection. **b** Schematic representation of fluorometric and colorimetric detection of the DEVD-dependent protease activity. The fluorometric assay detects the alteration in fluorescence emission of AFC after cleavage from the DEVD and the colorimetric assay detects the pNA after cleavage from the DEVD. **c** GST-P positive preneoplastic cells showing cleaved caspase-3 positive apoptotic cells. (A) GST-P positive hepatocytes showing cytoplasmic staining. (B) 1—Arrow pointing towards a hepatocyte with intense nuclear and cytoplasmic staining. 2—Condensed chromatin-containing caspase-3 positive apoptotic body [77]. **d** Specific primers are used to amplify the coding regions of p53 RNA derived from tumour, which are known to be the target of mutations. The amplified product is then introduced into an indicator yeast. The status of the p53 gene can be confirmed conferring to the color of yeast colonies. The mutant yeast with deficient adenine metabolism induces accumulation of P-ribosyl-amino-imidazole (AIR) metabolite, which turns red upon oxidation and promotes the formation of red colonies. **e** Schematic illustration of DSBs labelled with Br-dUTP utilising exogenous TdT

Activated caspases are among the biomarkers of programmed cell death. Cytokeratin 18 (CK 18, M30) is a caspase-cleaved protein and one of the biomarkers of apoptosis induced by chemotherapy [72]. During apoptosis, intermediate filament proteins (including CK18) are targeted for rapid breakdown by activated caspases 3, 7 and 9 to promote the creation of apoptotic bodies. Cytokeratin is formed into intermediate filaments of epithelial cells under normal physiological conditions and remains insoluble. A pool of soluble and insoluble cytokeratin (CK 8, 18 and 19) is also present in proliferating tumour cells, which may increase in response to stress [73]. de Hass et al. conducted the first clinical study on testicular cancer where the group used M30 and M65 ELISA in combination. M30 measures CK18 produced during apoptosis and M65 measures the level of caspase-cleaved and un-cleaved CK18. M30 and M65 are primarily based on specific sandwich ELISA technique combined with the antibody, where the circulating ccCK18 is quantified. M30 Apoptosense assay in which M30 is a monoclonal detection antibody also termed as M30 neoantigen, identifies neo-epitopes of CK18 (CK 18-Asp396) that are exposed only after caspase-cleavage of protein and it is postulated as a selective biomarker of apoptosis. While M65 is a probe of full-length and caspase-cleaved protein epitope and utilises M5 antibody as a catcher, thus measuring both forms of protein produced in response to apoptosis and necrosis (Fig. 5a) [72]. A high M30:M65 ration corresponds to the induction of apoptosis while a low M30:M65 ratio corresponds to necrosis induction [10]. Another type of sandwich ELISA is used to quantify released mature IL-1β as a result of activated caspase-1 during pyroptosis. Mature IL-1 β is released by activation of NLRP3 inflammasomes, which further activates caspase-1. Caspase-1 proteolytically activates the inflammatory cytokine IL-1 β and IL-18. In this technique, the immunoassay microplate containing processed cells is coated with monoclonal anti-IL-1 β antibody and blocked by blocking buffer. In the next step, IL-1β is captured through diluted recombinant IL-1 β standard and incubated overnight. In the final step, captured IL-1 β is detected with the help of detection antibody and chromogenic substrate and is measured through a plate reader [40].

### Fluorometric and colorimetric assays

The family of caspase of proteases [interleukin-1β-converting enzyme (ICE)/cell death protein-3 (CED-3)] has been identified as a key player in apoptosis. The family of human caspase consist of 10 proteases including caspase 1–10 and all have the ability to cleave substrate after aspartate residue. Likewise, caspase-3 is considered a mediator of apoptosis. Active caspase-3 cleaves poly ADP-ribose polymerase (PARP); an enzyme involved in DNA repair, genome integrity and surveillance. The cleavage of PARP by caspase-3 occurs at the onset of apoptosis and inhibition of PARP cleavage diminishes apoptosis. The activity of caspase-3 can be quantified through synthetic tetrapeptide, DEVD (a distinct amino acid sequence of Asp-Glu-Val-Asp), which is either labelled with a fluorescent molecule of 7-amino-4-trifluoromethylcoumarin (AFC) or with a colorimetric molecule of p-nitroanilide (pNA) (Fig. 5b). The DEVD-dependent protease activity indicates apoptosis and validates the effectiveness of the assay for assessing the role of the caspase family of proteases in apoptosis [74]. Through mapping of the cleavage site of PARP, the DEVD has been identified as a consensus site for caspase-3 cleavage [75]. The DEVD-dependent protease activity plays an important role in initiating apoptosis because it blocks apoptosis-mediated cell shrinkage and DNA fragmentation. The assay is performed by using commercially available kits and caspase activity is measured through a fluorescence plate reader or spectrophotometer at 380 and 405 nm, respectively, depending on substrate conjugating with the DEVD. If the DEVD is conjugated with AFC, it emits blue light on proteolytic cleavage. This assay can be used for rapid quantification of caspase-3 activity in the onset of apoptosis as it is a convenient, highly sensitive, speedy, and straightforward approach for detecting apoptosis [74]. Förster resonance energy transfer (FRET) is another fluorometric probe to distinguish between apoptotic and necrotic cell death. FRET is usually constructed in a vector gene, which plays the role of a reporter gene. FRET consists of donor fluorophore cyan fluorescent protein (CFP) and acceptor yellow fluorescent protein (YFP) joined with an activated caspase-specific amino acid linker DEVD in between. After caspase activation, the fluorescent proteins are physically separated and diffuse apart. Caspase activation is visualised by loss of FRET upon cleavage of the FRET probe. Retention of mitochondrial fluorescence and loss of FRET probe before its cleavage confirm necrosis. Since mitochondrial permeabilisation and subsequent caspase activation are prominent features of apoptosis, this method forms an extremely sensitive tool to visualise and quantify apoptosis and necrosis [10, 76]. Lekshmi et al. (2017) developed neuroblastoma cells, U251 expressing caspase sensors. The group observed single-cell colonies with a homogenous expression of the probe through an automated fluorescence microscope. Single-cell excitation for CFP and dual emission of YFP were collected in a ratio mode. Cells with activated caspase are easily distinguished with an increase in donor fluorescence and a decrease in acceptor fluorescence (CFP/FYP ratio). 10–15% of cells treated with doxorubicin lost fluorescence and depicted morphologic features indicative of necrosis such as enlarged cell size, vacuolisation, membrane alterations and so on. Lost solubility of genetically encoded FRET probe suggested changes in cell permeability associated with necrosis. FRET technology allows easy, fast and reliable discrimination between apoptosis and necrosis [76].

### Immunohistochemical methods

Antibodies have been proven a useful tool to recognise specifically cleaved form of various caspases including caspase-3 and 9. In 2004, Eckle et al. [77] tested the utility of antibodies that specifically identify large subunits of cleaved of caspase-3 and 9 through the immunohistochemical detection method. The group examined apoptosis in hepatocytes of rat liver sections. Cyproterone acetate (CPA), which blocks the apoptosis of hepatocytes was used as a negative control. H&E staining was done to observe apoptotic bodies. The cells were fixed and treated with rabbit-anti-cleaved caspase-3 or 9 antibodies. They performed a double immunostaining method which enabled the detection of caspase-3 positive apoptotic hepatocytes in preneoplastic liver lesion positive for glutathione-S-transferase-P (GST-P), which is abundantly expressed in mammalian tissue associated with malignancies. The double immunostaining method enabled the detection of apoptotic bodies stained by H&E, which were found correlated with a strong reduction of cleaved caspase GST-P positive hepatocytes. The staining of preneoplastic appeared in bluish colour and caspase-3 positive apoptotic cells appeared in red colour as shown in Fig. 5c [77]. Bonnet [78] developed an immunohistochemical protocol to distinguish between necroptotic and apoptotic cell death in the human epidermis. The protocol involves immunohistochemical staining against active caspase-3 revealed with a horse radish peroxidase (HRP) substrate 3,3 diaminobenzidine (DAB), followed by counter staining with H&E. DAB is a chromogen, which is used to visualise peroxidase activity. Oxidation of DAB produces brown precipitates, which permanently stains positive tissues. The apoptotic cells appeared caspase-3 positive using rabbit anti-active caspase-3 polyclonal antibody and exhibited brown staining while necroptotic cells appeared as dysmorphic, eosinophilic, caspase-3 negative dying cells. Another research group performed an immunohistochemical study on a high-grade astrocytoma to detect the activity of caspases. Human and mouse monoclonal antibodies were used to perform immunostaining. The results showed high-intensity staining depicting the presence of activity of caspase-3,6, 8 and 9 [78].

### Laser and mass spectroscopic methods

Detection of caspase activity on a single-cell level is a highly sensitive measurement. Dual-colour fluorescence cross-correlational spectroscopy (dcFCCS) is used to detect caspase activity when the concentration of substrate is very low (nanomolar). The apoptotic process in Jurkat cells was observed by fluorescence correlation spectroscopy (FCS) and caspase activity was investigated. The process of apoptosis was distinguished by the FCS curve that was different for non-apoptotic and apoptotic cells. D2R fluorescent probe was used which is a caspase substrate and the cleaved fluorescence indicated the presence of apoptotic cells [7].

Kohl et al. [79] utilised the dcFCCS technique with the combination of FCS, which is a minimally invasive technique to study molecular interactions, dynamics, temporal evolution, and monitor proteins in real-time. Protease activity through dcFCCS was studied in vitro by establishing green and red auto-fluorescent proteins. The assay completely relied on fluorescent proteins as they were connected by caspase-3 sensitive and insensitive proteins linker to create a dual colour protease substrate. After the expression of fusion proteins, dcFCCS was deployed for live/online (temporal) non-invasive analysis of intracellular protein stability and protease reactions. For concurrent fluorescence excitation of green and red auto-fluorescent proteins, two laser lines were overlaid which were coupled into an inverted microscope via a dual-band dichroic mirror positioned within the microscope and directed onto the object. The low number of dual-colour fusion proteins (intracellular reporter molecules) successfully increased the detection sensitivity for cellular processes and imitated native cellular physiological conditions more closely [79].

Self-assembled monolayers for matrix-assisted laser desorption ionisation time-of-flight mass spectrometry (SAMDI-MS) is also used to study caspase activity and apoptosis. Su et al. [80] performed label-free SAMDI-MS to measure endogenous caspase protease activities in cell lysates. The assay was based on the enzymatic modification of immobilised peptides on monolayer substrates, followed by direct detection of the product. Peptide substrate (presenting monolayer) for caspase-3 or 8 was treated with lysates from Jurkat cells and B-lymphoblastoid cell line (SKW4.6) that were stimulated with staurosporine and LzCD95L (leucine zipper FasL), respectively. SAMDI-MS reported the activation of endogenous caspase enzyme without the loss of enzyme activity with a similar level of detection, which is achieved by common fluorogenic assays [80].

## p53 activity detection methods

p53 is a nuclear phosphoprotein that plays a key role in the cellular response to DNA damage by inducing apoptosis or cell cycle arrest at the G1 phase. p53 protein plays a crucial role as a transcription factor and inducer of apoptosis and its disruption contributes to tumour progression and chemoresistance. p53 promotes apoptosis through transcription-dependent and independent mechanisms. Inactivation of wild-type p53 by the interaction of cellular or viral protein or by mutation has been found as a major reason for human cancer. p53 consists of four domains which are transactivation domain, central DNA binding domain, tetramerization domain, and regulatory domain. Mutation in p53 germline is associated with Li-Fraumeni Syndrome (LFS), which is a rare dominantly inherited cancer predisposition syndrome. Patients with LFS depict an increased risk of developing a wide range of cancer types [81]. Detection of p53 protein and mutation in p53 germline are achieved by the following methods.

### FASAY

Functional analysis of separated alleles in yeast (FASAY) is a yeast-based functional assay that detects the transcriptional activity, which is an essential function for tumour-suppressing activity of wild-type p53. p53 binds to a consensus sequence present in the promoter region of its target gene by its core sequence specific to the DNA binding domain. All known mutations are caused by a mutation in the DNA binding domain. p53 consensus binding sequence is located in the ribosomal gene cluster (RGC) and it is the sequence that is utilised in the FASAY assay [82]. FASAY is a colorimetric assay that is highly sensitive, semi-quantitative and specific for detecting mutation of p53, where wild-type p53 appears in white colonies and the mutant appears in red. Camplejohn et al. (2001) tested two rapid assays of p53 functions, namely apoptotic assay and FASAY to detect germline p53 mutation. FASAY detected mutations in exons 4–10 and provided no false-positive or negatives although apoptotic assay gave one borderline result without a mutation. FASAY not only offers the possibility of detecting p53 mutations but also characterises the mutation in terms of the two most important functions of p53 proteins, which are the induction of apoptosis and transactivation of the target gene [83]. Principally, the ADE2 gene of yeast encodes phosphoribosyl-amino-imidazole carboxylase (Ade2p) enzyme, which is involved in the purine biosynthesis pathway. The absence of Ade2p interrupts the synthesis of purine and causes the accumulation of the precursor P-ribosyl-amino-imidazole (AIR), which turns red upon oxidation and promotes the formation of red yeast colonies. Whereas sufficient level of Ade2p keeps the purine synthesis smooth and the yeast colonies appear in white as shown in Fig. 5d. ADE2 gene is placed under the control of a promoter composed of p53 response element RGC and fused to minimal CYC1 promoter. Lack of induction of transcription from p53 RGC response element leads to the to growth of red colonies and white colonies and vice versa. Intermediate amounts of Ade2p lead to pink colonies and indicate limited transcriptional activity of p53 [84]. As human p53 cDNA polymerase chain reaction (PCR) products are cloned directly in the reporter yeast strain through homologous recombination without intermediate bacterial cloning steps, the percentage of red yeast colonies correctly indicates the mutant p53 present in the starting material [85].

### p53 protein analysis method

Under most circumstances, a mutation in p53 gene correlates with the accumulation of p53 protein. The detection of accumulated protein is done immunohistochemically (IHC) or by ELISA, which are quantitative methods of detecting p53 protein. Levesque et al. [86] performed both IHC and ELISA for the detection of p53 proteins in lung tumor tissue. For IHC, the tissue was fixed in paraffin and washed with methanol peroxide. Microwave antigen retrieval was performed prior treating the tissue with biotinylated goat anti-mouse monoclonal antibody, which was washed by horseradish peroxidase-conjugated streptavidin. The slides were then counter-stained with hematoxylin and observed under a light microscope. Immunostaining was present in malignant cells and the nucleus was observed predominantly stained. Cells with faint cytoplasmic staining were considered negative for p53 protein. Sandwich ELISA was performed to compare the IHC results. Soluble p53 proteins present in lung tumor extracts were diluted in buffer and immobilised in microtiter wells coated with monoclonal DO-1antibody, which recognises same epitope on the surface of p53 proteins. The plate was then incubated and treated with polyclonal CM-1 antiserum and with alkaline phosphatase-conjugated goat antirabbit immunoglobulin. Fluorescence was measured at 615 nm by immunoanalyser. Concentrations of p53 protein in lung tumor extract were expressed relative to the total protein content because DO-1 antibody recognises the same epitope which is present at the amino-terminal domain shared by all conformations of p53 proteins [86].

## DNA fragmentation/denaturation/condensation detection methods

DNA fragmentation occurs due to the activation of the caspase cascade. DNA fragmentation begins from the activation of endonucleases and destruction of nuclear proteins. Activation of endonucleases cleaves chromosomal DNA at internucleosomal sections. Fragmentation of DNA is one of the main biochemical hallmarks of programmed cell death. Detection of DNA fragmentation during programmed cell death is achieved by following methods.

### APO ssDNA assay

APO ssDNA assay exploits the antibody produced against single-stranded DNA (ssDNA) to find damaged DNA in apoptotic cells [87]. This assay is focused on selective denaturation of DNA using formamide in apoptotic cells (a mild agent that denatures DNA only in apoptotic cells) and detection of denatured DNA by using ssDNA monoclonal antibody. The specificity of the assay is based on the high sensitivity of DNA condensed apoptotic chromatin in the presence of formamide to thermal denaturation, which is sufficient to generate large amounts of ssDNA at low temperature in the presence of formamide. In apoptotic and necrotic cells, strong denaturing agents, such as hydrochloric acid, induce DNA denaturation and are not appropriate for the staining of apoptotic cells [88]. The assay involves treatment of the cells attached in 96-well plates with formamide and one-step staining of the denatured DNA with a mixture of primary antibody and peroxidase-conjugated secondary antibody [87, 89]. APO ssDNA is a sensitive and specific assay for the identification of apoptosis.

### TUNEL assay

Fragmentation of DNA is demonstrated by the presence of multitude of DNA strands breaks and it is considered as a gold standard for the identification of programmed cell death. DNA fragmentation to 180–200 bp and more than 50 kbp is considered as a feature that clearly distinguishes apoptosis [71]. TUNEL (Terminal deoxynucleotidyl transferase (TdT)-mediated d-UTP Nick End Labelling) assay is used to detect DNA strand breaks (DSBs) in situ by labelling them with fluorochromes. Not only d-UTP but variety of deoxynucleotides, tagged with fluorochromes directly or indirectly, are being used in different types of TUNEL assay. TUNEL assay identifies and quantifies the apoptotic and necroptotic cells by fluorescence cytometry and microscopy. The method is based on template-independent identification of blunt ends (3’OH ends) of DSBs by TdT [90]. The histological section is pre-treated with protease, nick end labelled with biotinylated dUTP, and TdT then stained with avidin-conjugated peroxidase [9]. Instead of biotin or digoxygenin, the use of Br-dUTP as a conjugate with TdT increase the sensitivity of the assay and offers fourfold higher signals [36]. The process involves fixation of cells with formaldehyde, which prevents the extraction of DNA fragments by crosslinking to other cell constituents. The blunt end of DSBs serves as primers and becomes labelled with BrdU when incubated with Br-dUTP by a reaction catalysed by TdT exogenously. The labelled fragments can be immunohistochemically detected by Br-dU antibody conjugated with FITC and this detection does not require denaturation of DNA as shown in Fig. 5e [90]. Bivariate analysis of DSBs versus DNA material enables apoptotic subpopulations to be distinguished from nonapoptotic cell subpopulations and demonstrates the distribution of the cell cycle within those subpopulations [91]. Kelly et al. [92] induced cell death in LLC-PK cells to observe apoptosis and necrosis through TUNEL assay. The cells were fixed with paraformaldehyde and then stained with TUNEL reagent. The cells were stained with PI earlier to discriminate apoptotic and necrotic cells and TUNEL staining did not alter PI staining. Distribution of TUNEL staining among necrotic and apoptotic cells was observed through confocal microscopy and green TUNEL staining primarily labelled apoptotic cells. However, TUNEL staining was completely absent in necrotic cells. The group also observed apoptotic cells with secondary necrosis that were TUNEL negative and somewhere positive as well. Therefore, the model showed 99% specificity of TUNEL assay and 64% sensitivity (35% apoptotic cells were TUNEL negative, including apoptotic cells with secondary necrosis). The group studied another model of injured LLC-PK cells to observe apoptotic and necrotic cells. The models included H_2_O_2_ (a predominantly necrotic injury) or stuarosporine (a predominantly apoptotic injury) exposure as well as antimycin A-induced chemical anoxia followed by 24-h recovery. In all these models, PI was well retained after fixation. The appearance of necrotic TUNEL-positive cells (false positive) was observed predominantly in H_2_O_2_ model, which exhibited reduced specificity of TUNEL reaction in the predominantly necrotic model of cell injury. Conversely, the sensitivity of the TUNEL reaction was improved when applied to predominantly apoptotic models of cells. In summary, TUNEL reaction showed high degree of specificity exceeding 87% in all injury models except H_2_O_2_. All TUNEL positive cells were counted as true positive because of condensed and fragmented nuclei observed through fluorescent microscopy. Apoptotic cells undergoing secondary necrosis are the basis of confusion regarding specificity of TUNEL assay. The nonspecific activation of endonucleases during necroptotic cell death is believed to produce free DNA ends that could probably result in a TUNEL-positive reading. Apoptotic cells which escaped TUNEL staining depicted lack of high sensitivity of TUNEL assay, and it can underestimate apoptosis. The reason can be differences in DNA degradation among various apoptotic cells and/or the initiation of different apoptotic pathways in different models or injury [92]. The main mode of DNA cleavage differs between apoptotic and necrosis, i.e., nick in single strands of a double-stranded DNA molecule in necrosis and double-stranded DNA breaks in apoptosis [11]. Kraupp et al. [93] studied the specificity of TUNEL assay in well-defined models of apoptosis and necrosis in rat liver. Rat liver cells were induced by hepatomitogen to cause hyperplasia. After 48 h of repeated administration, upsurge of apoptotic bodies and regression of hyperplasia were observed. Chromatin condensation at nuclear membrane gives a clear-cut positive staining in TUNEL assay. The percentage of TUNEL-positive apoptotic bodies well corresponded with apoptotic bodies with condensed chromatin. Necrosis was induced in rat liver cells by CCl_4_ treatment and after 12 h and 24 h numerous vacuolised, lytic hepatocytes and ballooned hepatocytes were observed positive in TUNEL assay. The possible reason of high number of stainable DNA ends can be ladder-like DNA degradation or release of Ca^2+^ from intracellular stores or through injured cell membrane and subsequent activation of endonucleases. It has been observed that early stage necrotic cells demostrate highre levels of 3′-OH/5′P than 5′-OH/3′-P, and at late stage, this ratio revesred. This indicates that some different endonucleases get activated during necrosis, in addition to lysosomal dioxyribonucleases, which cause random DNA degradation [11]. The results of intervention performed by Kraupp et al. demonstrated that insufficiently preserved tissue or autopsy material may give false-positive results in test of apoptosis by TUNEL assay [93]. Tamura et al. [94] studied specificity of TUNEL assay by using immersion fixed heart tissues from both control rats and rats with heart failure. The heart specimens were divided into new and old immersion fixation groups with 2- and 8-weeks immersion fixation, respectively. The results showed significantly greater number of TUNEL positive nuclei of old immersion fixation group than new immersion fixation group. There was no significant difference of in number of TUNEL positive nuclei between normal and heart failure rats in both fixation groups. The group concluded that the specificity of the TUNEL method depends on the way the tissue is processed and the results of TUNEL staining should be processed with caution when immersion fixation is used and fixed tissues should be assayed by the TUNEL method before DNA degradation [94].

### ISEL

The in-situ end labelling technique (ISEL) detects apoptotic and necroptotic cells and labelled cells in which DNA has not fragmented yet and progressed to a nucleosome [95]. ISEL is a modification of the TUNEL assay introduced by Wijsman in 1993 [96]. The technique involves radioactive and non-radioactive labelling of free ends of DNA. Instead of TdT, ISEL is based on the activity of DNA polymerase 1 or (Kelnow fragment of DNA polymerase 1) that fills digitoxigenin tagged or biotinylated nucleotide substrates with broken 3’ends of DNA strands serving as a template in situ to synthesise new DNA fragments that can be visualised by a microscope after histochemical processing and enzymatic incorporation of labelled nucleotides at the sites of fragmented DNA can be detected by flow cytometry [9]. ISEL technique appears positive not only for apoptotic cells but also for necrotic cells. ISEL technique is used to quantify the dead cell in any tissue or cell culture [11].

### ELISA

Activation of endonucleases generates nucleosomal DNA, which is a biochemical hallmark of apoptosis however, it has been observed that specific form of DNA fragmentation also occurs during necrosis as an early event but appears to be triggered by proteolytic mechanism significantly different from apoptosis. Fragmentation of DNA occurs soon after loss of plasma membrane integrity. Additionally, serine proteases have been found involved in necrotic DNA fragmentation rather cysteine proteases, which suggest diverse pathway of endonuclease activation during cell death [97]. Endonucleases (caspase-activated DNase, CAD) cleaves chromosomal DNA into nucleosomes [98]. Two molecules of histones 3 and 4 form a central tetramer flanked by two dimers of histone 2A and 2B. This histone octamer is wounded by two super helical turns of DNA which makes a histone core particle. The next nucleosome particle is linked like a bead on a string of histone-free linker DNA. A molecule of histone H1 is located at the point where DNA comes in and leaves the nucleosome [99]. Fragmentation of DNA releases the histones (H1, H2A, H2B, H3 and H4) and it can be detected by ELISA. Nucleosomal ELISA is a sensitive but not apoptosis-specific method of detecting DNA fragmentation unless differential centrifugation method is also used to select for apoptotic DNA fragmentation. Specificity of nucleosomal ELISA depends on the antibodies utilised to perform assay [100]. Double antibody sandwich ELISA specifically recognises nucleosomes by a pair of monoclonal antibodies. Cytoplasmic nucleosomes captured from cell lysate onto an ELISA plate already coated with primary monoclonal antibody LG112, which reacted with the epitope of H2B. The bound nucleosomes were then detected by secondary antibody, biotinylated PL2-3 (specific for DNA and H2A-H2B dimer) and a streptavidin–alkaline phosphate conjugate. The apoptotic and necroptotic cells were clearly visible on ethidium bromide agarose gel [101].

### Gel electrophoresis-based methods

#### DNA ladder assay

**Fig. 6:**
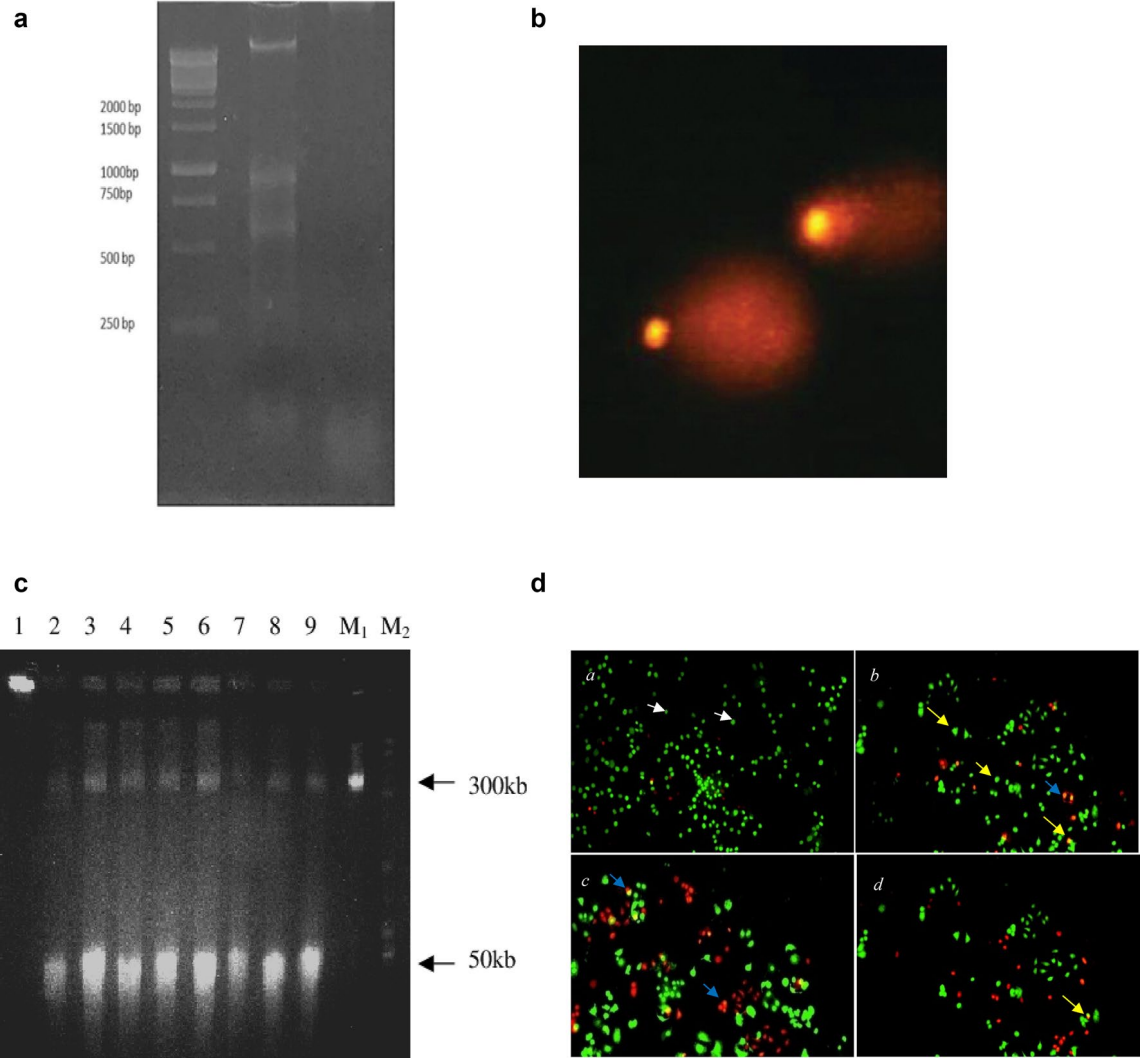
**a** 1.5% Agarose gel electrophoresis of DNA extracted from HNPMI treated and untreated cells MCF-7 cells. Lane 1—1 Kb Ladder, Lane 2—DNA of cells treated with HNPMI, Lane 3—DNA of untreated cells [39]. **b** SCGE showing a comet shape describes the amount of DNA in the nucleus as a head and the pattern and amount of DNA that has drifted away from the nucleus creating tail. **c** FIGE-separation of DNA from PHA-M-stimulated lymphocytes: preincubated without genotoxic agents (lane 1), preincubated with HP (0.05 mM, 20 min.), (lane 2), preincubated with HP (0.2 mM, 20 min.) (lane 3), and preincubated with HP (0.2 mM, 20 min.), then cultured with antimutagens: AN 100 uM (lane 4), AN 200 uM (lane 5), FPh 10 uM (lane 6), TDR 200óM (lane 7), AR 100óM (lane 8), AR 50óM (lane 9). M1, M2 Ą molecular weight markers. Adapted from [110]. **d** Fluorescence microscopy images of AO-EtBr staining for the detection of apoptosis in MCF7 cells (a) Untreated cells (b) MCF7 cells treated with 59.10 μM HNPMI (c) 64.10 μM (d) 69.10 μM. The appearance of green fluorescence (white arrow) in untreated control cells represents viable cells with normal morphology whereas visualisation of bright yellow-green colour (yellow arrow) and reddish yellow/orange staining (blue arrow) in the treated cells shows the presence of early and late apoptotic cells. Necrotic cells appear as a uniform orange stain [39]

DNA fragmentation occurs in two stages during apoptosis. DNA starts degrading sequentially. The first stage of fragmentation produces high molecular weight DNA fragments of 50–300kbps. In the second stage, a relatively smaller size of fragments containing 180–200 bps are produced. This stage of fragmentation occurs at the internucleosomal level and leads to the appearance of mono- and oligonucleosomes [99]. The extraction of DNA is done by chloroform-isoamyl alcohol and iso propanol and then oligonucleosomes are separated from the extracted DNA on 1.5% agarose gel and visualised after ethidium bromide staining (Fig. 6a). The nucleosomal ladder is characteristically seen as “DNA ladder” [8]. Necrosis progresses in rapid non-specific cleavage of DNA, while apoptosis activates endonucleases, which cleave DNA into fragments of approx. 180–200 bp. This is why the extracted DNA from necrotic cells looks like a shear and from an apoptotic cell, gives a ladder pattern. It is well known that in agarose gel electrophoresis, if the sample contains very few apoptotic cells, there is no demonstration of DNA ladder [11]. Moreover, DNA ladder assay is generally accepted as an apoptosis-specific technique because it detects oligonucleosomal cleavage rather artificial DNA cleavage or necrosis [100]. This method is used to separate low molecular weight DNA fragments which appear in a ladder pattern. Such a pattern of DNA degradation serves as a marker of cell death by apoptosis, and it is an apoptosis specific method to cell death detection [102].

#### Pulse field gel electrophoresis

Formation of DSB is a key feature of apoptosis called chromosomal DNA fragmentation. Nevertheless, some apoptosis inducers exhibit DNA damage induced DSBs prior to induction of apoptosis. Apoptotic DSBs appear as smeared low-molecular weight (less than 500 kb), while damage-induced DSBs result in a compact single band (more than 500 kb) [103]. Pulse field gel electrophoresis (PFGE) is a powerful technique for the separation of large DNA molecules ranging from a kilo to mega base pairs after digestion of a unique restriction enzyme. In this technique, cells are encapsulated in agarose to form cell plugs, thus preventing any morphological damage to the cells while placing them in agarose gels where electrophoresis will take place. PFGE has been reported to be very sensitive to several factors which affect the migration of DNA, including the pulse time, the electric field, temperature, agarose concentration, angle between the fields and field geometry [104]. Principally, the intact chromosomal DNA is isolated by lysing cells embedded in an agarose plug to avoid mechanical clipping of molecules during extraction. After extraction, digestion of the chromosomal DNA within the agarose plug by a rare cutting restriction enzyme occurs which produces high molecular weight DNA fragments. The digested fragments of DNA (10–800 kb) are then exposed to alternative electric fields between spatially distinct pairs of electrodes for separation. This process facilitates mega-base size DNA fragments (from 200 to over 12,000 kb) to reorient and migrate at different speeds towards the anode in a size dependent manner through gel pores. This process renders a good determination of large DNA fragments in agarose gel and helps in distinguishing apoptotic and non-apoptotic DNA fragmentation [105].

#### Single-cell gel electrophoresis (comet assay)

Single-cell gel electrophoresis (SCGE) is a microelectrophoretic assay is used to detect DNA fragmentation as a result of radiation or hyperthermia. This method helps in visualising the DNA damage at a single-cell level [106]. Damaged cells are suspended in melted agarose gel, lysed in a neutral detergent and then cast on a microscope slide. The electric field of 5 V/cm is applied for 5 min and then staining is done with a fluorescent dye. It is also known as a comet assay because of the migration pattern of DNA which appears like a comet on electropherogram (Fig. 6b) [107]. A produced comet can characterise the amount of DNA in the nucleus as a head, and the pattern and amount of DNA that has drifted away from nucleus creating a tail embedded in thin-layer agarose gel during electrophoretic separation [108]. The intensity of the tail is proportional to the amount of damaged DNA which has migrated from the head to tail region [9]. By fluctuating the pH of electrophoresis, various forms of DSBs can be detected. Under alkaline conditions, ssDSBs, dsDSBs, excision repair site and alkaline-labile site can be detected. However, under neutral conditions, it mainly detects dsDSBs and therefore, it is considered suitable for detecting apoptotic cells [109].

#### Field inversion gel electrophoresis

Field inversion gel electrophoresis (FIGE) is based on the periodic inversion of a uniform electric field in one dimension, principally. This method was derived from orthogonal-field-alternation gel electrophoresis (OFAGE). Application of higher voltage or longer time of one-dimension electric field results in net migration of DNA fragments or proteins than in opposite direction. FIGE allows the separation of DNA or proteins mixtures in size ranges not available to regular electrophoresis (Fig. 6c). Because of the homogeneous electric field, the DNA migrates in a straight lane, making lane-to-lane comparison simple and reliable. FIGE has been used successfully to separate linear DNA molecules with low, medium, high, and very high molecular weight, ranging several hundred bases to at least 6 Mbp. Aside from these benefits, the FIGE technique allows for separation over a wide range of molecular weights at low resolution or selection of a small “window” with relatively high resolution. Also, the integrity of the DNA molecule that weights up to 2Mbp can be analysed by FIGE, which is a unique feature of this technique [34]. FIGE is a non-apoptosis specific technique as it detects a wide range of fragmented DNAs with lowest to high molecular weight. In summary, if the different zones of high resolution, compression and band inversion are clearly defined with known size standards, this method has some very appealing resolution power. Once calibrated, FIGE can be very effective for screening large amounts of samples, such as yeast artificial chromosomes [110].

#### DNA-specific fluorochrome based methods

DNA fragmentation and extensive loss can be determined by measuring DNA content using intercalating dyes such as acridine orange, ethidium bromide, propidium iodide or dyes which, get bind to DNA externally like Hoechst 33342, 4′-6-diamino-2-phenyl indole (DAPI), YO-PRO-1 and mithramycin [111]. The activation of an endonuclease during programmed cell death leads to the extraction of low molecular weight DNA following cell permeabilisation, which consequently decrease the staining ability of cells with DNA-specific fluorochromes [112]. The process of DNA condensation particularly occurs during apoptosis while necrosis does not indicate any event like DNA condensation. Hoechst 33342 is a bis-benzamide derivative dye used to observe nuclear condensation, which binds to AT-rich sequences in the minor grove of double-stranded DNA groove [113]. When excited by ultraviolet light, this dye fluoresces at 461 nm and can therefore be visualised using traditional fluorescent microscopes [114]. When DAPI was used to detect control cells in ISEL, the cells showed a low level of staining due to impermeability of cell membrane to nucleotide triphosphate however, when DAPI was used with apoptotic cells in ISEL stronger and greener signals were observed as compared to the control cell [34]. On the other hand, acridine orange (AO) is a fluorescent dye, which can easily cross the cell membrane and it is accumulated in the lysosome. AO is a weak base, which gets absorbed in organelles due to their low pH. When inside, the dye becomes protonated as a consequence of the high proton concentration and is eventually entrapped in these organelles. Low-AO concentration supravital cell staining, which appears as red fluorescence, is a result of the activity of the lysosome proton pump [115]. In fact, AO accumulation is mainly determined by the pH gradient across the lysosome membrane that is maintained by the proton pump based on ATP. The monomeric and polymeric binding of AO to the cellular DNA results in green and red fluorescence, respectively as shown in Fig. 6d. Green fluorescence can decrease during apoptosis due to DNA breakdown, while red fluorescence is not altered because lysosomes' ability to accumulate AO remains intact during the initial stages of apoptosis [36]. Other dyes and their mode of action are summarized in Table 2.

**Table 2:** Common dyes for DNA staining

S. No.	Dyes	Permeability	Emission/fluorescence	Binding site
1	DAPI	Cell permeable	461/Blue	A-T selective
2	Propidium iodide	Cell impermeable	538/Red	DNA intercalator
3	Acridine orange	Cell & organelle permeable	525,651/Green	DNA intercalator/Lysosomes
4	Hoechst 33342/33258	Cell permeable	461/Blue	A-T selective
5	Ethidium bromide	Cell impermeable	605/UV 300 nm	dsDNA, RNA
6	YO-PRO-1	Cell impermeable	509/Green	DNA, RNA
7	Red-DotTM 1	Cell permeable	694/Far-red	DNA
8	NucSpot 470	Cell impermeable	546/Green	DNA
9	NucSpot 650	Cell permeable	675/Far-red	DNA
10	TMRM	Organelle permeable	574/Orange	Mitochondria
11	Rhodamine 123	Organelle permeable	529/Green	Mitochondria
12	DRAQ5TM	Cell permeable	665/Far-red	A-T minor grove
13	SYBR Green	Cell permeable	520/Green	DNA intercalator

## Comparative analysis

Various methods of detecting cell death have evolved to identify and discriminate the damaged cells from healthy cells, and to observe the effect of a therapeutic agent which is given to cancerous or unhealthy cells to undergo cell death. In this review, the mechanisms and mode of detection of various conventional and non-conventional methods of cell death have been critically reviewed and in this section, a comparative analysis has been drawn categorically to identify the advantages and limitations of reviewed methods.

Morphological manifestations of cell death involving shrinking of cells, membrane blebbing, swollen cytoplasm, increased cytoplasmic density, pyknosis which can be observed under light microscopy by following a routine staining method. H&E staining stains nuclear chromatin fragments in dense purple color and the cytoplasm in dark eosinophilic color of apoptotic cells [34]. Hematoxylin staining shows different cell-type- and cancer-type-specific patterns of condensation of heterochromatin, which are diagnostically very important, while eosin stains nucleoli. The cytoplasm of an apoptotic cell appears in a distinct shade of blue, if numerous polyribosomes are present. The Golgi zone can be normally identified by the absence of staining in a region near the nucleus [116]. Light microscopy is an inexpensive method of detecting apoptosis however, it is prone to error and requires expertise. The reproducibility and objectivity of this method are also very low because it detects the low number of apoptotic cells. As compared to light microscopy, electron microscopy outlines subcellular changes better. It displays the most obvious changes like extensive plasma membrane blebbing, condensation of chromatin and its peripheral aggregation under the nuclear membrane, and separation of fragments of cells into apoptotic bodies by budding. Electron microscopy allows the observation of fine ultrastructural changes associated with cell death, such as gaps in the plasma and/or the mitochondrial outer membrane, mitochondrial swelling, and the first stages of chromatin condensation (which only later become visible by light microscopy). Although electron microscopy can provide plenty of ultrastructural information, visual inspection of electron microphotographs should always be accompanied by a rigorous quantitative approach. Indeed, because the analysis is performed on a per-cell basis and only a subset of cells within each sample can be studied, it is critical for researchers to avoid focusing on rare (or even fictitious) morphologies. Furthermore, sample preparation/staining for electron microscopy is time-consuming and necessitates the use of trained personnel. Nonetheless, immunoelectron microscopy procedures can provide very useful information. Scanning electron microscopy (SEM) and transmission electron microscopy (TEM) are two facilities that can be utilized to study morphological changes associated with cell death. TEM is reputed as a “gold standard” in the field of cell-death research [117]. TEM offers higher resolving power (0.1–0.4 nm). In fact, it offers a two and three-dimensional image of the internal cell and allows the comprehension of biological structure–function relationships at (sub)cellular and molecular levels. For these reasons, TEM is considered the most accurate method for distinguishing apoptosis and necrosis in cell cultures however, it is a time-consuming, expensive, and laborious method to adapt for apoptosis detection.

In relation to microscopic techniques, Annexin V binding assay can be visualised by flow cytometry, light, and fluorescence microscopy. As compared to other dyes, PI, 7-AAD and trypan blue which are impermeable dyes, Annexin V detects the apoptosis while cell membrane is partially intact where other dyes are failed to detect onset of apoptosis and indicates the loss of viability of cells. However, Annexin V is specific for PS and shows minimal binding to other phospholipids like phosphatidylcholine, and sphingomyelin, which are constitutively present in outer the leaflet of the plasma membrane [36]. Another setback of Annexin V is to demonstrate a false-positive result by entering through the holes of necrotic cells and binding with PS, which makes it less reliable and makes the use of additional stain mandatory [8]. Availability of LDH in the extracellular space is also a biomarker of cell membrane damage and indicates apoptosis or necrosis. LDH is a good parameter to measure the percentage of damaged cells in the sample because of the integral linearity of this assay. LDH is a simple, accurate and reliable alternative to 51Cr release cytotoxicity assay, a radioactively labelled cytotoxicity assay, which is used to measure the low level of cytotoxicity. The LDH assay also detects low-level damage to the cell membrane, which cannot be detected by other methods. The release of LDH occurs at the early stage of necrosis but at a late stage of apoptosis, which is one of the setbacks of LDH assay as it cannot detect the onset of apoptosis and the detection of late apoptosis might be converted into necrosis [118]. However, measuring LDH is insufficient to classify cell death as pyroptosis. Caspase-1 dependence is necessary for distinguishing pyroptotic cell death from necrotic cell death or apoptosis followed by secondary necrosis (lysis of apoptotic blebs) [119]. Two unconventional detection methods of membrane damage and capacitance have been reviewed and both are noninvasive, non-destructive, rapid, cost-effective and simple to produce as many other spectroscopic and flow cytometric methods. DEP measures cell stress rather than cell death by detecting ion efflux early in the apoptotic process. However, the electrochemical methods are unable to measure apoptosis on single-cell level or deliver cell-specific information over a large cell population [7].

MTT and XTT assay based on oxidoreductase-driven reactions are widely used to examine cell viability and proliferation reliably. Nevertheless, tetrazolium dyes/salts have different reaction rates with superoxides. MTT and XTT assay can overestimate viability of cells or fail to detect a decrease in cell numbers and viability because of the concentration of superoxides or other molecules that also react with superoxide in the cell. These competitive reactions may undermine evaluations and interpretations of proliferation and viability of cells. In this context, tetrazolium dyes for cell viability and proliferation cannot be considered accurate and reliable if experimental conditions influence the level of O_2_-in biological system [120]. Cationic lipophilic fluorochromes have been extensively used to measure the functionality of mitochondria in numerous cells. These fluorochromes are permeable to the plasma membrane and are used to assess the changes in MMP. The ideal MMP-sensitive fluorochrome probe detects the alteration in MMP with a decreased uptake in the mitochondrial matrix. The behaviour of the fluorochromes may depend on environmental factors (phototoxicity and susceptibility to local variations) independent of MMP. Some fluorochromes are observed highly toxic and interfere with the bioenergetic function of mitochondria. Some fluorochromes undergo self-quenching upon accumulation in the mitochondrial matrix, some show non-specific interaction with thiol or lipids, some get influence by multidrug resistance-associated proteins and some depend on plasma membrane potential magnitude [121]. Among the four classical fluorochromes ((DiOC6(3)), TMRE, JC-1, CMX-Ros), JC-1 is considered as the best fluorochrome which measures alteration in MMP with great accuracy in intact cells because it produces two fluorescence emission peaks that reflect the existence of two forms of the dye. An ideal probe suitable for MMP determination must exhibit maximum resolution between high and low peaks of MMP. JC-1 monomers emit predominant green, fluorescent light at low MMP and red–orange fluorescence (aggregated JC-1) at high MMP. Whereas, DiOC6(3) responds to both, changes in MMP and plasma membrane potential however, it may alter membrane conductivity and may inhibit energy metabolism of the cell, which consequently disturbs the MMP and may display false-positive result. Rh123 and TMRE passively distribute between cytosol and mitochondria and this transmembrane distribution of the dyes only depends on MMP [122]. High concentrations of fluorescence dyes to measure MMP are proven lethal for cells and saturate the binding sites as well. Rh123 and DiOC6(3) have been observed toxic to mammalian cells [123]. Changes in MMP have an immediate effect on ATP production because the synthesis of ATP is strictly related to MMP. Respiratory control is disrupted by uncouplers that dissipate proton gradient and induce maximal oxygen consumption without producing ATP. MASC assay for measurement of ATP synthesis in cultured mammalian cells is a sensitive, rapid, and convenient assay as compared to other conventional assays where cells are disrupted mechanically, and cell suspension is centrifuged before examination. However, MASC assay includes only several washes of the microplate and can be performed with 103 cells while other conventional methods require 106 cells. MASC assay provides a high-throughput analysis [64]. The releases of cytochrome c from mitochondria is a significant initial step in the activation of cell death pathways. The level of circulating cytochrome c represents mitochondrial injury. ELISA and western blot techniques are used to quantify cytochrome c release in the extracellular fluid from death-induced cultures of cell lines. Commercially available kits to perform ELISA are highly sensitive and selective but they usually carry time-consuming protocols, are expensive, require large sample volume, and demand expertise [66]. However, ECL-ELISA, which is an unconventional type of ELISA, displays a wide dynamic range by using a small volume of the sample. ECL-ELISA is a reproducible technique surpassing the performance of other viable techniques. Also, it minimises the need for sample dilution which makes it an easy and rapid technique [69].

M30 (caspase cleaved CK18) is a specific antibody that appears at the early stage of apoptosis and does not react with intact or necrotic cells hence detection of M30 is a reliable indicator of apoptosis and immunological staining with M30 antibody has shown correlation with other apoptosis assay like TUNEL, and ISEL [124]. The expression of M30 has been observed high in cancerous cells however, the M30 IHC assay does not detect apoptosis in epithelial-derived cancer cells and ELISA should be used in conjugation to avoid false-negative [125]. Colorimetric and fluorometric assay to detect caspase-3 activity through labelled-DEVD substrate is a quantitative assay. The assay provides rapid and consistent quantification, and it is also suitable for a time-course analysis of cultured cells. It can be performed with complex tissues with several types of cells where only one type undergoes to apoptosis. However, the assay requires homogenisation of the tissue for enzyme release which destroys the integrity of the tissue being examined and impedes localisation of the event within the tissue. Also, there are limitations regarding the size of tissue that can be used to assess caspase-3 activity through this assay [126]. Detection of caspase activity by the IHC method is a quantitative study that allows the counting of apoptotic bodies because cleaved caspase-positive apoptotic bodies are easily detectable and countable. Also, the caspase cleaved-negative apoptotic bodies are observable through H&E immunostaining. One big setback of this method is that it is highly time-consuming and only remainders of apoptotic cells are observable whereas, pre-apoptotic cells are very difficult to detect [77]. dcFCCS and SAMDI-MS are unconventional quantitative methods with high resolution for detecting caspase activity. dcFCCS provides a temporal assessment of caspase activity on single-cell level with minimal invasion. The technique facilitates the monitoring of dynamic equilibria in vivo under normal physiological conditions. dcFCCS tolerates the delayed maturation of red fluorophores, which is an insurmountable obstacle for fluorescence resonance energy transfer (FRET), which is a fluorescent technique to measure caspase activity. dcFCCS and SAMDI-MS provide better resolution than fluorescent assays, but both are expensive instruments and demand expertise [7, 79].

p53 tumour suppressor protein is a transcription factor that facilitates the cell during various kinds of stress by preventing cell division or by initiating apoptosis. Mutation in p53 gene has been identified as a reason for 50% of human cancers. Several methods have been developed to detect and analyse mutation in p53. IHC for detecting p53 protein is a rapid and simple technique and it can identify clinically relevant distinct staining patterns at single-cell resolution [127]. However, a major setback often observed of IHC method is the significant rate of false-negative and false-positive interpretations of the mutational state of p53 gene. Also, the fixative may affect the intensity of staining and distribution of stained cells labelled by a single anti-p53 antibody [128]. On the other hand, ELISA is far less subjective and precludes high level of professional training, which is required to interpret the results meaningfully in a standardized manner. Additionally, the requirement for p53 protein to bind two immunoreagents at the same time and multiple washing steps between incubation time contribute to increasing specificity ELISA greatly. On the contrary, ELISA requires fresh frozen tissues and cannot be performed with fixed tissues which is a major setback of ELISA [86]. Immunoblotting protocols are time-consuming, unsuitable for large-scale applications, and only provide reliable semiquantitative results when using primary antibodies at sub-saturating concentrations. Furthermore, while fluorescence-based detection provides higher sensitivity than traditional chemiluminescence, detecting small and/or weakly expressed proteins may be difficult and/or time-consuming. Finally, while (immuno)fluorescence microscopy-based quantifications are performed per cell, semiquantitative immunoblotting data represent whole cell populations, regardless of intrapopulation or intercell heterogeneity. As a result, immunoblotting is unsuitable for analysing heterogeneous cell samples such as primary tissues or solid tumors. Among all the classic transactivation assays, FASAY has been identified as the most effective and convenient method of detecting p53 mutation. All the other methods are based on either luciferase or β-galactosidase reporter genes, which are time-consuming and laborious methods involving cloning and co-transfection steps. FASAY has an advantage over other transactivation methods because of two principles: homologous recombinant-based DNA repair mechanism and transcription activity of p53 in yeast. FASAY is a simple, sensitive, and specific method of detection of mutation in p53 with the benefits of discriminating inactivating mutations from silent mutations and gene polymorphism [129].

One accepted and very important biochemical hallmark of programmed cell death is DNA fragmentation that results in a characteristic “DNA ladder” however, its absence does not eliminate the fact that cells are undergoing to cell death because fragmentation of DNA is a later event of the apoptosis process. DNA ladder assay is suitable for tissues or cell cultures with a high number of apoptotic cells per tissue mass but not ideal for those with low numbers [126]. As compared to DNA ladder assay, the ISEL technique identifies apoptotic cells and labelled cells prior to the progression of DNA fragmentation into precise nuclear fragmentation. ISEL is an extremely sensitive method because several avidin-FITC and biotinylated anti-avidin layers form biotin-avidin interaction which makes the immunofluorescent detection highly sensitive. One setback of the ISEL is detection of Okazaki fragments with free 3′-ends, which appear as fragmented DNA and are a false-positive result [130]. Although ISEL is a modified form of TUNEL assay for detecting fragmented DNA, TUNEL assay is the most popular and more sensitive method. TUNEL assay reactions are based on direct labelling of 3′-OH termini of DNA breaks and these breaks are detectable on a molecular level. The TUNEL assay can be used to detect the onset of apoptosis because it detects DNA breaks that occur at an early stage of apoptosis when apoptosis cannot be detected morphologically [34]. On the contrary, TUNEL is an expensive assay because of the high cost of enzymes and antibodies that are used in the assay. Furthermore, histological fixation and pre-treatment also affect the DNA strand break detection. A major setback is the detection of necrotic cells providing false-positive results of apoptosis. Sometimes, the TUNEL assay identifies cells in process of DNA replication and active gene transcription and provides false-positive results [131]. Another vital gel-based technique of DNA fragmentation detection is comet assay. The comet assay is a fast test to detect DNA damage. It is an easy, sensitive, precise, and quantitative assay. The comet assay not only detects DNA damage that occurs due to radiation like γ-rays and X-rays, but it has been found useful for monitoring extra-biomonitoring environmental pollution [132]. The comet assay has higher sensitivity than the TUNEL assay and the DNA ladder assay, as it provides specific information about the degree and heterogeneity of DNA damage. There are minor disadvantages of comet assay. Like other detection methods, it is also a time-consuming, multi-step, and tedious assay, which somewhat damages the cell membrane and changes the distribution of cell population of live, necrotic and apoptotic cells [34]. FIGE can currently be considered complementary to the other PFGE methods due to its unique resolution features. However, because this method is more sensitive to many parameters than the other pulsed-field techniques, it appears to be more difficult to use and has received less practice. Some electrophoretic conditions need to be improved to reduce band broadening while achieving maximum resolution over a wider size range, but it has proven to be very adequate for most sizing and screening applications. The advantages of FIGE are accompanied by the serious issue of band inversion, in which larger DNA molecules travel faster than smaller ones. Using FIGE, the mobility of DNA molecules as a function of molecular weight decreases as DNA size increases. However, it eventually reaches a minimum that, under certain conditions, can be close to zero. Surprisingly, DNA with a size greater than this minimum point has greater mobility. This is known as “band inversion”. Because of this effect, measuring the size of specific DNA bands can be difficult at times. It may be possible to shift the separation range so that the DNA size of interest is not affected by band inversion by using appropriate conditions. DNA-specific fluorochromes also identify apoptosis when the plasma membrane excludes its uptake. Dyes like PI, DAPI, Trypan blue, Hoechst, and AO are used to detect apoptosis. PI does not enter the cell at an early stage of apoptosis because of the intact cell membrane however, Hoechst 33342 dyed apoptotic cells appear brighter than control [133]. Detection of apoptosis through DNA-specific fluorochromes is an easy, rapid, accurate and quantitative method in both viable and fixed single cells but it is a multi-step process and for intact tissues, it requires pre-treatment of an enzyme to release individual cells for analysis [34]. A short summary has been presented in tabulated form describing pros and cons of popular assays/techniques (Table 3).

**Table 3:** Pros and cons of popular techniques/assays

S. No.	Assay/technique	Biomarkers/indicator	Instrument/device	Strength	Limitation
1	Light microscopy	Morphological changes	Light microscope	Inexpensive	Prone to error/Low in reproducibility
2	Electron microscopy	Morphological changes	Electron microscope (TEM, SEM)	Provides a high-definition field for observation	Expensive and time-consuming
3	Annexin V assay	Changes in plasma membrane	Light, fluorescence microscope/flow cytometer	Sensitive for early-stage apoptosis	Detects necrosis as well
4	Lactate dehydrogenase assay	LDH enzyme	Fluorescent microplate reader	Detects low-level cytotoxicity	Only detects late stage of apoptosis
5	Electrochemical methods	Changes in the plasma membrane/Cytoplasm conductivity	3DEP cell analyser, fluorescence microscope, signal generators	Inexpensive, easy	Does not provide information at single-cell level
6	MTT and XTT assay	Mitochondrial respiration	Fluorescent microplate reader	Reliable and convenient	Overestimates the cell viability under O-2 influence
7	Mitochondrial membrane potential assay	Changes in MMP/Depolarisation of transmembrane potential	Fluorescence microscope, flow cytometer	Permeable to the plasma membrane	Highly dependent on nature of the dye
8	MASC assay	ATP production	Microplate luminometer	Easy, convenient	Expensive
9	ELISA	APAF-1/activated caspase 1,2,3,7,8, and 9/cytochrome c/cytokeratins/p53/nucleosomal DNA	Photometer, flow cytometer	Highly sensitive and selective, reliable	Expensive, time-consuming, cell/tissue type-dependent
10	IHC methods	Caspase activity/Apo-1/Fas/Bcl-2/p53	Fluorescence microscope	Inexpensive	Time-consuming, laborious, often provides false-negative results
11	FASAY	p53 mutation	PCR/RT-PCR device	Simple, sensitive, and reliable	–
12	TUNEL assay	DNA fragmentation	Flow cytometer	Detects onset of apoptosis	Provides false-negative by detecting necrotic cells
13	ISEL	DNA fragmentation	Flow cytometer	Highly sensitive	Detects Okazaki fragments as DNA strand break
14	Comet assay	DNA fragmentation	Electropherogram/electrophoresis device	Highly sensitive	Time-consuming, multistep process

## Concluding remarks

There are plenty of methods for measuring cell death-related parameters that rely on various technologies and can be distinguished by their specificity, sensitivity, limitations, precision, and throughput. Each of these techniques was designed with a clear objective, and some have evolved to be more general in nature. As a result, a plethora of protocols for studying cell death are now available. Nonetheless, none of these methods is sufficient in and of themselves to prove cell death, and a combination of complementary but unrelated techniques should always be used. Such methodological obfuscation may result in the use of assays that are completely inappropriate for the experimental setting under investigation. Programmed cell death detection methods can be improved by developing and refining the current methods which can distinguish early and late apoptosis stages, secondary necrosis, can investigate temporal features of apoptosis, can provide qualitative analysis that can be utilised for quantification, can define apoptosis index to indicate prognosis and metastasis of cancer, and can decrease the chances of false-positive and false-negative results. To illustrate the involvement of the same biological phenomenon, at least two complementary but methodologically different approaches should be used to characterise cell death in mechanistic terms. Combining miniaturised fluorogenic assays with cytofluorometry and/or immunofluorescence microscopy-based testing, for example, can confirm caspase activation without a doubt. However, because cell death is very diverse, the signalling pathways that lead to cell death may differ even among experimental circumstances that are otherwise similar. As a result, it is up to each investigator to determine which biochemical parameters should be tracked for the mechanistic characterisation of cell death in his or her experimental setting. Nevertheless, accurate and precise interpretations of programmed cell death will be possible because continuous progress and developments are being made to understand the process of cell death which can bring about the development of new protocols to overcome the current challenges.

## Data Availability

Not applicable.
